# Targeting metabolic dependencies to reverse chemoradiotherapy resistance in colorectal cancer

**DOI:** 10.1186/s13046-026-03755-x

**Published:** 2026-06-23

**Authors:** Maximilian Hellkamp, Simon Gertken, Jonas Buchloh, Julius-Leonard Hellwig, Lukas Ben Kowitzke, Ningjun Duan, Ilaria Gaspardo, Stefan Küffer, Michael Linnebacher, Philipp Ströbel, Matthias Wirth, Volker Ellenrieder, Stefan Rieken, Jochen Gaedcke, Michael Ghadimi, Jürgen Wienands, Günter Schneider, Melanie Spitzner, Marian Grade

**Affiliations:** 1 Department of General, Visceral and Pediatric Surgery, University Medical Center Göttingen, Göttingen, Germany; 2 Research Training Group (RTG) 2978, University Medical Center Göttingen, Göttingen, Germany; 3 Institute of Pathology, University Medical Center Göttingen, Göttingen, Germany; 4 Molecular Oncology and Immunotherapy, Clinic of General Surgery, University Medical Center Rostock, Rostock, Germany; 5 CCC-N (Comprehensive Cancer Center Lower Saxony), Göttingen, Germany; 6 Department of Hematology, Oncology and Cancer Immunology, Charité - Universitätsmedizin Berlin, corporate member of Freie Universität Berlin and Humboldt-Universität zu Berlin, Berlin, Germany; 7 Department of Gastroenterology, Gastrointestinal Oncology and Endocrinology, University Medical Center Göttingen, Göttingen, Germany; 8 Department of Radiotherapy and Radiooncology, University Medical Center Göttingen, Göttingen, Germany; 9 Clinic for General and Visceral Surgery, Städtisches Klinikum Karlsruhe, Karlsruhe, Germany; 10 Institute of Cellular and Molecular Immunology, University Medical Center Göttingen, Göttingen, Germany; 11 Institute for Translational Cancer Research and Experimental Cancer Therapy, Technical University Munich, Munich, Germany

**Keywords:** rectal cancer, chemoradiotherapy, MCT1, GLUT1, metabolism, glycolysis, therapy resistance

## Abstract

Neoadjuvant chemoradiotherapy (CRT) constitutes a standard treatment for locally advanced rectal cancer (RC), frequently followed by radical surgical resection. Yet, therapeutic responses vary widely, and intrinsic radioresistance remains a major barrier to cure. To uncover actionable determinants of CRT response, we established a panel of patient-derived colorectal cancer cell lines (PDCLs) followed by integrated phenotypic and functional characterization. We identified metabolic reprogramming as a hallmark of radioresistance and, through orthogonal validation experiments, confirmed elevated glycolytic and mitochondrial ATP production in (chemo)irradiation-resistant PDCLs. The causative relationship of this association and its potential for therapeutic intervention was shown by subsequent drug screening, showing resistance to most of the applied drugs and revealing a critical dependency on the monocarboxylate transporter (MCT1) and the glucose transporter 1 (GLUT1). Metabolism-targeting compounds re-sensitized resistant PDCLs to irradiation; especially inhibition of GLUT1 exhibits a robust radiosensitizing activity across models. Concordantly, GLUT1 expression correlated with poor response to neoadjuvant CRT in our own RC patient cohort and various publicly available patient datasets. Collectively, our study defines metabolic dependency as a key driver of CRT resistance in RC, and reveals glycolysis- and lactate-transport-associated pathway activities as targetable vulnerabilities. These findings provide a mechanistic basis for patient stratification and support the development of metabolism-directed strategies to overcome (chemo)radioresistance in RC.

**Supplementary Information:**

The online version contains supplementary material available at 10.1186/s13046-026-03755-x.

## Introduction

Approximately one third of colorectal cancers (CRC) are located in the rectum. Globally, the incidence of rectal cancer (RC) is rising, with projections estimating one million new annual cases by 2040, 10% of which will occur in adults under 50 [1, 2]. About half of all patients present with locally advanced, mismatch repair-proficient rectal cancer (LARC), for which the standard treatment frequently involved neoadjuvant chemoradiotherapy (CRT) followed by total mesorectal excision [3–6]. To further improve outcomes and to increase the fraction of patients with a complete clinical response – enabling organ-preservation and sparing patients from radical surgery (via the watch-and-wait strategy) – total neoadjuvant therapy (TNT) has emerged, which administers all systemic chemotherapy and radiotherapy prior to surgery [1, 7–12]. However, responses to CRT and TNT are highly variable, and treatment failure is commonly associated with impaired prognosis, underscoring a critical unmet need for predictive biomarkers and novel therapeutic strategies to personalize treatment and improve survival [1, 10, 11, 13, 14].

Resistance to radiotherapy is a multifactorial phenomenon driven by the interplay of tumor-intrinsic aberrations, the tumor microenvironment (TME), and metabolic adaptation [15–17]. Known contributors to therapy resistance of RC patients include genetic alterations such as *TP53*, *APC*, *KRAS* mutations, microsatellite instability, dysregulated signaling pathways (e.g., PI3K or DNA damage signaling), and epigenetic alterations [18]. Our group identified additional mediators of therapeutic resistance including components of the IL-6/JAK/STAT3 pathway, the WNT/β-catenin axis, and the NOTCH signaling cascade [19–21].

To further identify actionable therapeutic vulnerabilities of CRT, we now used a panel of 14 primary patient-derived cancer 2D cell lines (PDCLs), derived from RC patient tissue, CRC patient-derived xenografts (PDXs), or CRC patient-derived organoids (PDOs), and comprehensively characterized their morphology, growth kinetics, and mutational landscapes. Using integrated live-cell imaging and endpoint viability assays, we stratified these models into a (chemo)irradiation-resistant (IR-resistant) group and a (chemo)irradiation-sensitive (IR-sensitive) group. To identify resistance dependencies, we performed a focused drug screen revealing broad resistance to conventional chemotherapeutics but heterogeneous susceptibility to targeted agents. Mechanistically, IR-resistant cell lines and RC patients exhibited metabolic dependencies, with enriched pathway activity in both glycolysis and oxidative phosphorylation. Consequently, we demonstrated efficacy of metabolic inhibitors specifically in IR-resistant models, unveiling a targetable metabolic vulnerability to overcome CRT resistance.

## Materials and methods

### Cell culture of patient-derived cell lines and SW1463

**Fig. 1:**
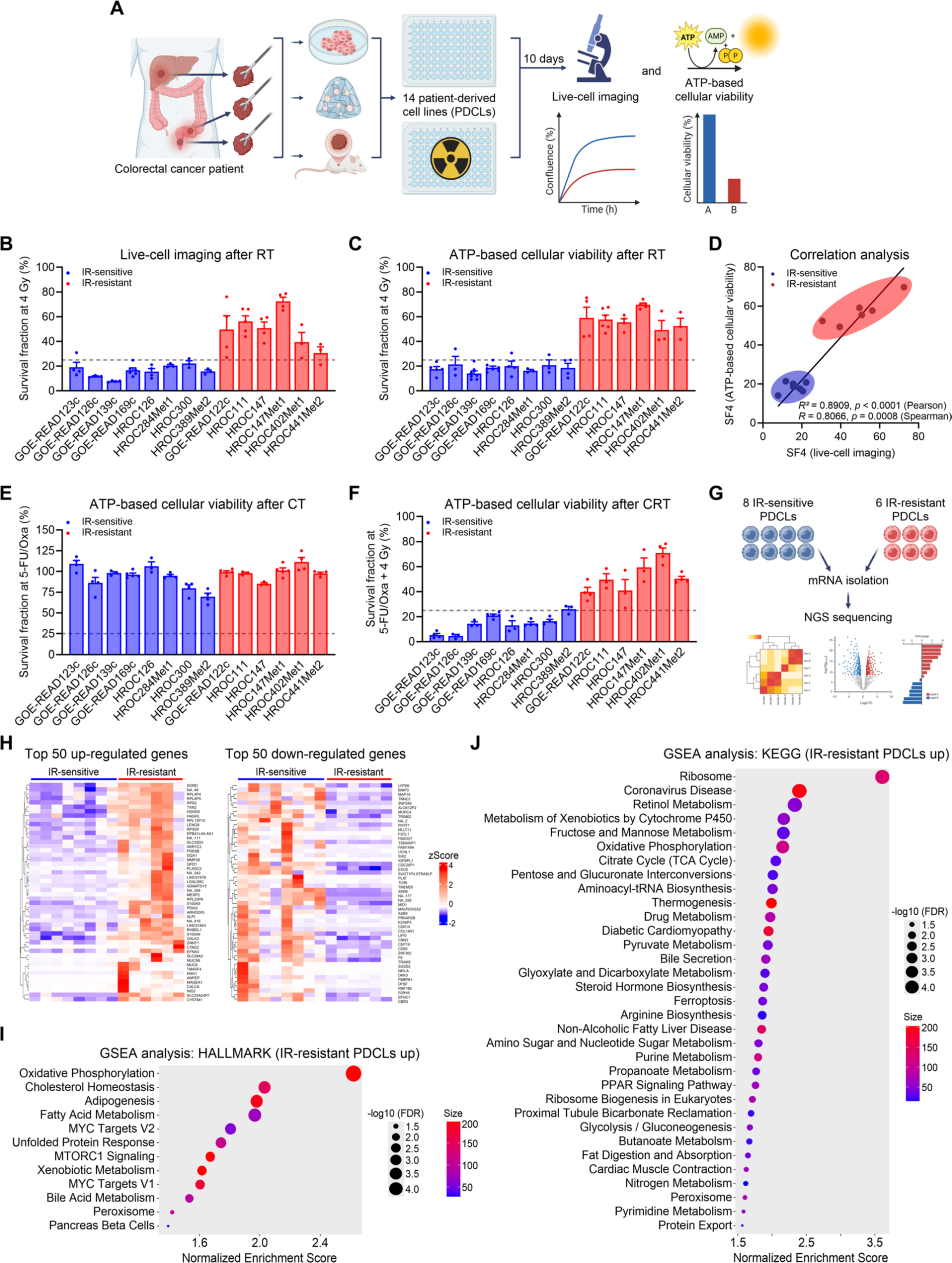
Heterogeneous irradiation sensitivity across a panel of 14 patient-derived cell lines. **A** Schematic illustration of the three sources of patient-derived cell lines (PDCLs): direct derivation from primary tumor tissue, patient-derived organoids (PDOs), and patient-derived xenografts (PDXs) and the experimental workflow for irradiation assays, including daily confluence monitoring over ten days and ATP-based cellular viability assessment. **B-C** Survival fractions at 4 Gy (SF4) determined by live-cell imaging (**B**), ATP-based cellular viability assays (**C**). Colors denote irradiation (IR)-sensitive (blue) and IR-resistant (red) cell lines. **D** Correlation analysis between SF4 values derived from live-cell imaging and ATP-based cellular viability assays. **E**-**F** Survival fractions of 3 µM 5-Fluorouracil / 1 µM Oxaliplatin-based chemotherapy (CT, **E**), SF4 of chemoradiotherapy (CRT, **F**). Colors denote (chemo)IR-sensitive (blue) and (chemo)IR-resistant (red) cell lines. **G** Workflow for mRNA analyses. **H** Heatmap depicting differentially expressed genes in IR-sensitive vs. IR-resistant PDCLs. **I**-**J** Gene set enrichment analysis (GSEA) comparing IR-sensitive and IR-resistant cell lines using the HALLMARK gene sets (**I**), KEGG gene sets (**J**). PDCL, patient-derived cell line; SF4, survival fraction at 4 Gy; IR, (chemo)irradiation; CRT, chemoradiotherapy; GSEA, gene set enrichment analysis

The panel of PDCLs comprised a total of 14 cell lines (Fig. 1A). Nine cell lines (HROC111, HROC126, HROC147, HROC147Met1, HROC284Met1, HROC300, HROC389Met2, HROC402Met1, and HROC441Met2) were obtained from the University Medical Center Rostock as part of the patient-derived xenograft HROC biobank [22]. Five PDCLs (GOE-READ122c, GOE-READ123c, GOE-READ126c, GOE-READ139c, and GOE-READ169c) were derived from corresponding patient-derived organoids (PDOs), which were previously established using surgical specimens obtained from CRC patients at the University Medical Center Göttingen (UMG) [23]. Briefly, after mechanistically fragmentation of the organoids, cells were seeded into cell culture-coated 12-well plates (#83.3921; Sarstedt, Nürnbrecht, Germany) and maintained in Dulbecco´s Modified Eagle Medium/Nutrient Mixture F-12 (DMEM/F-12; #D8437; Sigma, Darmstadt, Germany), supplemented with 10% fetal bovine serum (FBS; #TMS-013‐B; Merck Millipore, Berlin, Germany), 1% penicillin/streptomycin (P/S; #15140‐122; Gibco, Schwerte, Germany), and 2 mM L-glutamine (#25030‐081; Gibco), in a humified atmosphere at 37 °C and 5% CO_2_. An isogenic IR-resistant SW1463 cell population (SW1463_RES) was established from ATCC-obtained SW1463 (RRID: CVCL_1718; ATCC, Manassas, VA, USA) by repeated IR-doses of 2 Gy (total of 68 Gy), and control cells (SW1463_PAR) were cultured for the same amount of passages in parallel to the irradiated cells as previously described [20]. During cell culture, media changes were conducted every 48–72 h, and passaging was performed in logarithmic growth phase with confluences between 70% and 80%. All cell lines were routinely tested for mycoplasma using MycoAlertVR Mycoplasma Detection Kit (#LT07-318; Lonza, Basel, Switzerland). In addition, all cell lines were authenticated using short tandem repeat (STR) profiling (Multiplexion GmbH, Friedrichshafen, Germany). All experiments were carried out using cells that had undergone fewer than 20 passages after thawing from frozen stock. Further information of cell line characteristics can be found in Fig. S1A and Table S1.

### DNA panel sequencing

DNA from all PDCLs was isolated using DNA extraction kit QIAamp^®^ DNA Mini Kit (#51306; Qiagen, Hilden, Germany) and sent for DNA panel sequencing to the Institute of Pathology (UMG). DNA sequencing was performed by a QIAseq targeted DNA custom panel (Qiagen) of 26 genes (205 amplicons), which are commonly mutated in CRC (Table S2). For each cell line, 10 ng of isolated DNA was amplified with four customized GeneRead Primer Pools (Qiagen). PCR products from the same patient were pooled after treatment with FuPa reagent (#A26435; Thermo Fisher Scientific, Life Technologies, Waltham, MA, USA). Following purification with Agencourt AMPure XP (Beckman Coulter, Brea, CA, USA), PCR products were incubated with NEXTflexTM DNA Adenylation Mix (Bioo Scientific, Austin, TX, USA). NEXTflexTM DNA Barcodes were used as adapters (Bioo Scientific). After bead size selection, NEXTflexTM PCR Master Mix (Bioo Scientific) was used for the final PCR amplification. Library products were quantified with a Qubit 2.0 Fluorometer (Qubit ds DNA HS Kit, Life TechnologiesTM), diluted and pooled in equal amounts. 6–8 pM were spiked with 5% PhiX DNA (Illumina, San Diego, CA, USA) and sequenced with the MiSeqTM reagent Kit V2 (300-cycles; Illumina). Data were exported as FASTQ files. FASTQ files were aligned against reference NCBI build 37 (hg38) on the in-house pipeline [24]. Resulting mutational status of the PDCLs is summarized in Table S2.

### Quantitative monitoring of cell growth via live-cell imaging

First, the optimal seeding density was determined for each cell line by plating 250-4,000 cells per well in transparent (#83.3924; Sarstedt, Nümbrecht, Germany) and white 96-well plates (#136102; Thermo Scientific, Roskilde, Denmark) and incubated for ten days at 37 °C with a medium change on days four and seven. Morphology and proliferation were monitored using the Celigo^®^ Imaging Cytometer (RRID: SCR_018808; revvity, Hamburg, Germany) on days four, seven, and ten. Viability was assessed on day ten using CellTiter-Glo^®^ (CTG) Luminescent Cell Viability Assay (#G7573; Promega, Walldorf, Germany) as an endpoint measurement in the VICTOR™ X4 2030-0040 Multilabel Plate Reader (RRID: SCR_025714; PerkinElmer Cellular Technologies Germany GmbH, Hamburg, Germany). Data from both readouts were analyzed to define the final seeding density of each cell line for subsequent experiments (optimized cell numbers can be found in Table S3a-b). For cell growth monitoring, cells were seeded in three technical replicates into clear 96-well plates (Sarstedt) and placed into an Incucyte^®^ SX5 Live-Cell Analysis Instrument (RRID: SCR_026298; Sartorius AG, Göttingen, Germany). The instrument was set to the following; scan type: adherent cell-by-cell; objective: 10x; image channel: phase. Four images per well were acquired every 12 h for ten days with a medium renewal every four days. Proliferation was quantified as confluence using the AI confluence mode of the Incucyte^®^ Analysis Software (Incucyte 2024 A; Sartorius). Detailed Incucyte^®^ analysis parameters are summarized in Table S3a. Doubling time was derived during the logarithmic growth phase from confluence values (N) at 24 h (t_1_) and 144 h (t_2_) using the following formula:

$$\:Doubling\:time\:\left[h\right]=\:\frac{\left({t}_{2}-{t}_{1}\right)\cdot\:\mathrm{ln}\left(2\right)}{\mathrm{ln}\left(\frac{{N}_{2}}{{N}_{1}}\right)}$$

### Irradiation assays

Three independent methods were used to characterize the irradiation sensitivity of the PDCLs. To determine the confluence and for cellular viability assays, cells were seeded at their optimized density (Table S3a-b) in three technical replicates into two clear 96-well plates (Sarstedt). For CRT experiments, cells were allowed to adhere for eight hours, followed by treatment with 3 µM 5-Fluorouracil (5-FU; #6627; Sigma-Aldrich, Taufkirchen, Germany) and 1 µM Oxaliplatin (#O9512; Sigma-Aldrich, Taufkirchen, Germany). After 24 h, one plate was irradiated with 4 Gy (XCELL^®^225; Kubtec; Stratford, CT, USA), while the control plate was sham-irradiated. Eight hours after irradiation, a media change was conducted to remove chemotherapy (CT). The plates were imaged in the Incucyte^®^ SX5 (Sartorius) according to the experimental setup described above. For analysis, the survival fraction at 4 Gy (SF4) was calculated as ratio of the confluence values from the irradiated plate versus the sham-irradiated control plate on day ten after irradiation. As a second method, cellular viability was analyzed by the CellTiter-Glo^®^ Luminescent Cell Viability Assay (Promega) on the last day of the incubation period. To this end, cell lysates were transferred into white 96-well plates (Thermo Scientific) and luminescence values were determined by the VICTOR™ X4 2030-0040 Multilabel Plate Reader (PerkinElmer). Survival fraction at 4 Gy was calculated as ratio of the luminescence values from the irradiated or the CRT plate versus the sham-irradiated control. To discover the effect of the chemotherapy on the PDCLs, the ratio of the luminescence values from the CT-treated plate versus the control plate was calculated.

Colony formation assays were conducted as previously described [21]. Briefly, cells in logarithmic growth phase were seeded as single-cell suspensions into 6-well plates (#83.3920; Sarstedt). For cell numbers, see Table S3c. Adherent cells were pre-treated with 3 µM 5-FU (Sigma-Aldrich) for 16 h prior to irradiation and subsequently irradiated with single X-ray doses of 0, 0.5, 1, 2, 4, and 6 Gy followed by cell line-specific post-irradiation incubation periods (Table S3c). Finally, colonies were fixed with 70% ethanol (#2212.9025; ChemSolute TH Geyer, Renningen, Germany), stained with Mayer´s hemalum solution (#1.09249.2500; Merck KGaA, Darmstadt, Germany) and counted. Colonies consisting of more than 50 cells were scored as survivors. Plating efficiency was determined and surviving fractions were normalized to non-irradiated controls [25].

### Drug screening

For drug screening, cells were seeded in 96-well plates (Sarstedt) at cell densities previously established to achieve approximately 75% confluence after 96 h (Table S3d). Twenty-four hours post-seeding, cells were treated with a library of 126 compounds (TargetMol Chemicals Inc., Boston, MA, USA) using a pin tool (#AFIX96FP; V&P Scientific, San Diego, CA, USA), as previously described [26]. Compound concentrations were generated via 3-fold serial dilution (10 µM as highest concentration), with seven concentrations per drug. Cellular viability was assessed 72 h post-treatment using CellTiter Glo^®^ Cell Viability Luminescent Assay (Promega). After adding 25 µl of the reagent and a 20-minutes incubation on an orbital shaker, luminescence was measured using a VICTOR™ X4 2030-0040 Multilabel Plate Reader (PerkinElmer). Data analysis was performed using the R package GRMETRICS [27], with dose-response curves analyzed by calculating the mean area under the curve (meanAUC). All AUCs are reported in Table S6.

To identify compounds active in irradiation-resistant cells, the deltaAUC was calculated as the difference between the meanAUC of irradiation-resistant and irradiation-sensitive cell lines for each drug, where negative deltaAUC values indicate enhanced activity in irradiation-resistant cells.

### Drug screen validation and determination of IR-sensitization scores

To achieve high precision and data reproducibility in applying five drug dilutions, we used the robot-assisted liquid dispenser MANTIS^®^ (FORMULATRIX^®^, Dubai, UAE). For these experiments, we selected six PDCLs (GOE-READ122c, GOE-READ126c, GOE-READ139c, HROC111, HROC284Met1, HROC441Met2) as well as SW1463_PAR, and SW1463_RES. For each cell line, two white 96-well plates (Thermo Scientific) were seeded using individual cell numbers that have been previously established (Table S3e). Following cell attachment, four drugs per plate were applied at five concentrations each using MANTIS^®^ (FORMULATRIX^®^). On the next day, one plate was irradiated (Kubtec) with either 2 Gy (IR-sensitive cells) or 4 Gy (IR-resistant cells). Medium was changed with renewal of the drug dilutions on day four. Seven days after seeding, cellular viability was assessed by CellTiter-Glo^®^ Cell Viability Luminescent Assay (Promega) using the VICTOR™ X4 2030-0040 Multilabel Plate Reader (PerkinElmer), and dose-response curves were generated. An irradiation-sensitization score (ISS) was derived in analogy of the Highest Single Agent (HSA) model [28]. For ISS determination, first, inhibition values of the single drug doses and the applied irradiation dose were calculated to the untreated control from cellular viability values. Next, the inhibition of the combined treatment was calculated for each drug dose and the higher single inhibition value (either drug, or irradiation) was subtracted to obtain an HSA-equivalent value for each dose. Finally, the mean ISS was calculated with positive values indicating radiosensitization, and negative values indicating enhanced radioresistance.

### RNA isolation

For RNA sequencing (RNA-seq) analyses, total RNA was isolated from two million PDCLs grown in T25 flasks (#83.3910.002; Sarstedt) at 80–90% confluence. Additionally, for our analysis, we incorporated the isogenic IR-resistant SW1463 model (SW1463_PAR/SW1463_RES) [20], which had been used in a previous RNA-seq study from our group (unpublished data). SW1463_PAR/SW1463_RES cells undergone RNAi-treatment, but only control-siRNA-transfected cells (siNEG; QIAGEN) were included in this analysis. For transfection, one million cells were seeded into 6-well plates (#83.3920; Sarstedt), and RNA was isolated after 48 h. To investigate the effect of the MCT1 inhibitor AZD3965 on mRNA expression levels by quantitative RT-PCR (qPCR), RNA was collected from six PDCLs (GOE-READ122c, GOE-READ126c, GOE-READ139c, HROC111, HROC284Met1, HROC441Met2). Cells were allowed to adhere overnight and then treated with either DMSO or 10 µM AZD3965 (MedChemExpress; # HY-12750, Monmouth Junction, NJ, USA) for 72 h. Subsequently, total RNA was isolated for downstream analysis. For RNA isolation, cells were harvested, washed with PBS, and pelleted by centrifugation at 500 g for five minutes at room temperature. The pellets were lysed in 350 µl RLT buffer (#79216; QIAGEN), and total RNA was isolated using the RNeasy Mini Kit (#74104; Qiagen) according to the manufacturer´s instructions. RNA quantity was determined using a NanoDrop^®^ ND-1000 Spectrophotometer (RRID: SCR_016517; Thermo Fisher Scientific Inc., Waltham, MA, USA), and samples were adjusted to 100 ng/µl. Purified RNA was stored at -80 °C until further analysis.

### Quantitative RT-PCR (qPCR)

PCR was performed using the GoTaq^®^ 1-Step RT-qPCR System (#A6020; Promega) on a BIO-RAD CFX384TM Real-Time PCR Detection System (BIO-RAD, Hercules, CA, USA) as previously described [19]. The medians of the resulting cycle threshold (C_t_) values were normalized to the housekeeping gene *HPRT1*, and relative gene expression changes of *SLC16A1* (MCT1) and *SLC16A3* (MCT4) upon AZD3965 treatment were calculated according the 2^−ΔΔCT^ algorithm [29]. For primer sequences see Table S3h.

### RNA sequencing and data processing

RNA sequencing was performed at the Next Generation Sequencing (NGS) – Integrative Genomics Core Unit (NIG), Institute of Pathology, UMG. RNA quality was assessed by measuring the RNA integrity number (RIN) using a Fragment Analyzer HS Total RNA Kit (#DNF-472-FR; Agilent Technologies, Santa Clara, CA, USA). Library preparation for RNA-seq was performed in the STAR Hamilton NGS automation using the Illumina Stranded mRNA Prep (#20040534; Illumina) and the ID for Illumina RNA UD Indexes Set A, ligation with 96 indexes (#20091646; Illumina) starting from 200 ng of total RNA. The size range of the final cDNA libraries was determined by applying the SS NGS Fragment 1–6000 bp Kit (#DNF-473-1000; Agilent) on the Fragment Analyzer (average 320 bp) (Agilent). Accurate quantification of cDNA libraries was performed using the DeNovixDS-Series System (DeNovix Inc., Wilmington, DE, USA). cDNA libraries were sequenced using an S1 flow cell Illumina NovaSeq 6000 Sequencing System (RRID: SCR_016387; Illumina); 100 cycles, 20 Mio reads/sample.

Sequence images were transformed with BaseCaller Illumina software to BCL files and demultiplexed to fastq files with bcl2fastq v2.20.0.422. Sequencing quality was determined using FastQC v0.11.5 software [30] (RRID: SCR_014583). RNA-seq QC used FastQC with 32 threads and MultiQC to aggregate reports [30–32]. Reads were aligned to GRCh38 using STAR v2.7.8a with an index built from Ensembl (RRID: SCR_002344) release 107 GTF; options included: sjdbGTFfile Homo_sapiens.GRCh38.107.gtf, sjdbOverhang 50, outSAMtype BAM SortedByCoordinate, quantMode GeneCounts, outFilterMismatchNmax 2, and limitBAMsortRAM 3,000,000,000 [33]. Gene-level counts used featureCounts (RRID: SCR_012919) v1.5.0-p1 with paired-end mode and 32 threads (-p -P -T 32) against the same GTF, gene IDs and raw counts were extracted from the featureCounts Table [34].

### Differential gene expression analysis and gene set enrichment analysis

For analysis of the differential gene expression, DESeq2 (RRID: SCR_015687) v1.44.0 with independent filtering and BH correction was used, thresholds were adjusted to *p* < 0.05 and log2FC ≥ 1 with genes < 10 counts filtered. Plots used EnhancedVolcano (RRID: SCR_018931) v1.22.0, ComplexHeatmap (RRID: SCR_017270) v2.20.0, ggplot2 (RRID: SCR_014601) v3.5.2, and vsn (RRID: SCR_001459) v3.72.0 [32, 35]. Table S4 includes log2 fold changes (log2FC), *p*-values, and *p-adj*-values of the analyzed genes in PDCLs. This analysis was performed at the NIG (Institute of Pathology, UMG, Göttingen). For SW1463_PAR/SW1463_RES, rLog normalized counts were generated using the DESeq2 tool from the web-based Galaxy Europe application [36] (Table S8). Gene set enrichment analysis (GSEA) for PDCLs was processed on the GenePattern website [37] via the module GSEA v20.4.0. Analysis was performed on two gene set data bases, first KEGG (version 2021); second on cancer pathways from the Molecular Signatures Database (MSigDB, v2025; RRID: SCR_016863) HALLMARK collection (H, v2025) [38, 39]. Because sample size from one group was *n* < 7, permutation type was set to “gene_set” and the collapse dataset was set to “No_collapse”. Table S5 includes enrichment scores, *p*-values, and *q*-values of the gene sets in PDCLs.

### Genomic co-expression analyses and gene set enrichment analyses of patient cohorts

For co-expression correlation studies (Spearman; *SLC16A1* and *SLC2A1*), the entire Göttingen rectal cancer patient cohort [40] was analyzed. Data can be accessed via the NCBI Expression Omnibus (GSE87211). For publicly available data sets, mRNA expression data from two other studies were downloaded from cBioPortal [41–44] (RRID: SCR_014555), first the Rectal Cancer Memorial Sloan Kettering Cancer Center (MSK) cohort [45], and second, the rectal adenocarcinoma cohort from The Cancer Genomic Atlas (RRID: SCR_003193) (TCGA) / Genomic Data Commons Data Portal (GDC Data Portal; RRID: SCR_014514) [46]. For both cohorts, “samples with mRNA data” (MSK: Agilent microarray, 100 samples/patients; TCGA: RNA-seq TPM, 173 samples/patients) were chosen, and a co-expression analysis (Spearman’s correlation) for *SLC16A1* and *SLC2A1* was performed. Results of all three cohorts were subjected to a GSEA (MSigDB HALLMARK collection) via the web-based application GeneTrail [47] (v3.0; RRID: SCR_006250; Center for Bioinformatics, Saarbrücken, Germany). Table S9 includes co-expression results and enrichment scores, *p*-values, and FDR-values of the GSEA analysis.

### Assessment of clinicopathological characteristics in rectal cancer patients

Tumor regression grades (TRG) of the Göttingen cohort were evaluated using the Dworak classification system [48] and was recently described by Buchloh et al. [23]. A TRG of 4 was used as a surrogate for tumors highly responsive to neoadjuvant treatment (i.e., tumors with a complete histopathological regression), while a TRG of 1 indicates non-responsive tumors. Table S7 provides detailed clinicopathological characteristics of patients stratified by TRG, including TRG 1 and TRG 4.

### Metabolic profiling by Seahorse^®^ XF assay

Glycolytic activity and mitochondrial function were assessed by Seahorse^®^ XF Real-Time ATP Rate Assay (#100777-004; Agilent Technologies) using the Agilent Seahorse XFe24 Cell Analyzer (RRID: SCR_019539; Agilent Technologies). Cells were seeded at individual cell numbers (Table S3f) in Seahorse^®^ 24-well plates. After 72 h, oxygen consumption rates (OCR) and extracellular acidification rates (ECAR) were measured upon serial injection of 1.5 µM oligomycin and 0.5 µM rotenone/antimycin A as specified by the manufacturer. Following measurements, cell numbers for each well were determined by trypsinization and cell counting in a hemocytometer. For data analysis, the “.asyr” file containing the raw data was uploaded to Agilent Seahorse Analystics [49] (version 1.0.0-739) and a XF ATP Rate Assay analysis was performed. Raw data for basal rates of ATP production were cell number-normalized for each well resulting in MitoATP (pmol/min/1,000 cells) and GlycoATP (pmol/min/1,000 cells) values. Furthermore, kinetic graphs of cell number-normalized oxygen consumption rate (OCR, pmol/min/1,000 cells) and extracellular acidification rate (ECAR, mpH/min/1,000 cells) were generated.

### Lactate assay

Extracellular lactate levels in the cell culture medium were measured using the Lactate-Glo™ Assay (#J5021; Promega) according to the manufacturer’s instructions. Briefly, cells were seeded at their specific densities (Table S3g) in DMEM/F12 (Sigma), supplemented with 10% dialysed FBS (#FBS-DIA-12B; Capricorn, Ebsdorfergrund, Germany), 1% P/S (Gibco), and 2 mM L-glutamine (Gibco), and allowed to adhere overnight. On the following day, cells were treated with DMSO or 1 µM or 10 µM AZD3965 (MedChemExpress) and incubated for 72 h. Cell culture medium was collected, diluted 1:150 in PBS (#20012-019; Gibco), and 12.5 µl of each sample was transferred to a white 384-well plate (#781080; greiner, Frickenhausen, Germany). Subsequently, 12.5 µl Lactate Detection Reagent was added and the plate was incubated for one hour at room temperature in the dark. Luminescence was recorded using the multi-well plate reader Tecan infinite 200Pro (RRID: SCR_020543; Tecan Austria GmbH, Grödig, Austria). Luminescence signals were background-corrected, and the ratio of luminescence in AZD3965-treated samples relative to DMSO-treated controls was calculated.

## Results

### Heterogeneous irradiation and chemoradiotherapy response of PDCLs

Fourteen PDCLs were established from either direct seeding of single tumor cells of CRC patients into cell culture dishes, or indirectly from PDOs or xenografts (Fig. 1A) [22, 50]. Among the 14 patients providing tumor specimens for the generation of PDCLs, seven (50%) had received neoadjuvant therapy prior to surgical resection, with CRT in five patients and two patients with chemotherapy alone. All underlying patient information is summarized in Fig. S1A and Table S1. Panel sequencing (Fig. S1A, Table S2) showed that PDCLs carry cancer-type specific mutations such as *TP53* (93%), *APC* (86%), *KRAS* (57%), *POLE* (43%), and *SMAD4* (36%). Morphological characterization by microscopy revealed heterogeneous growth patterns, which included different morphological types like an epithelial growth type, and a more staged-like growth pattern (Fig. S1B). Live-cell imaging showed marked heterogeneity in proliferation dynamics across the PDCL panel. Two lines, GOE-READ139c and HROC284Met1, exhibited rapid growth with doubling times of approximately 20 h, whereas others displayed significantly slower proliferation, with doubling times exceeding 60 h (Fig. S1C-D, Table S1).

To evaluate the effects of irradiation and CRT across the lines, three complementary assays were performed: (i) standard colony formation assay (CFA; Fig. S2A-B, D), (ii) quantification of cellular confluence by live-cell imaging (Fig. 1B), and (iii) analysis of ATP-based cellular viability as a surrogate for growth (Fig. 1C, F, Fig. S2F). For CRT experiments, chemotherapeutic agents were used in a radiosensitization approach with overnight incubation prior to irradiation. In the CFA setting, we used 3 µM 5-FU, which is similar to the serum concentration of RC patients treated with 5-FU-based CRT [51]. Due to insufficient plating efficiencies of seven PDCLs, colony formation analysis was restricted to the other seven cell lines (GOE-READ139c, GOE-READ169c, HROC126, HROC284Met1, HROC300, HROC147, HROC147Met1). As a result, CFA analyses identified five IR-sensitive and two IR-resistant PDCLs (Fig. S2A-B, D). Interestingly, the addition of 5-FU did not further reduce survival fractions (SFs) across all seven cell lines (Fig. S2A, D).

From the live-cell imaging analyses (Fig. 1B) and the ATP-quantification of the cellular viability assays (Fig. 1C), similar results regarding the irradiation responses at 4 Gy were obtained. Correlation analyses of the SF4 (survival fraction at 4 Gy) derived from these seven PDCLs demonstrated strong concordance across all three methodologies (Fig. S2C, E), confirming that the 96-well plate-based experimental setup (Fig. 1A) recapitulates outcomes observed in the conventional CFA. Consequently, confluence monitoring and ATP-quantification were applied for the remaining seven PDCLs. Since RC patients are typically treated with neoadjuvant CRT based on the FOLFOX regimen consisting of folinic acid, 5-FU, and Oxaliplatin [3], we incorporated 3 µM 5-FU and 1 µM Oxaliplatin (IC_20_; Fig. S2G) as a pre-irradiation CT protocol (Fig. 1F). Notably, despite using concentrations in the micromolar range, the PDCLs exhibited remarkably high survival fractions exceeding 75% (Fig. 1E), indicating a substantial intrinsic resistance to this standard combined CT-based regimen.

All three approaches revealed a marked heterogeneity in radiation and CRT response at 4 Gy (Fig. 1B-C, F; Fig. S2B, D), which is not confounded by neoadjuvant treatment of patients (Fig. S1A) or cellular doubling time (Fig. S1D). Specifically, six PDCLs exhibited an IR-resistant phenotype, while eight demonstrated sensitivity to irradiation and CRT. Highly significant correlations (Pearson and Spearman’s, *p* < 0.001; Fig. 1D) were observed between SF4 values calculated from confluence measurements and cellular viability assays, validating the reproducibility of these high-throughput methods.

Next, we analyzed transcriptome profiles to elucidate molecular determinants of radiation resistance (Fig. 1G). After dividing the PDCL panel into IR-resistant and IR-sensitive groups, we identified a total of 3,135 significantly differentially expressed genes (DEGs; Table S4). The heatmap in Fig. 1H shows the top 50 up-regulated and down-regulated DEGs, and the volcano plot in Figure S3A displays the distribution of all DEGs across the entire dataset. Next, Gene Set Enrichment Analysis (GSEA) was conducted to gain a deeper understanding of the biological processes underlying radiation resistance. Using the HALLMARK and KEGG signatures, GSEA revealed significant enrichment of several gene sets in IR-resistant PDCLs compared to IR-sensitive cells (Fig. 1I-J, Table S5). Notably, in the HALLMARK signatures, cancer metabolism-associated gene sets, e.g., oxidative phosphorylation, fatty acid metabolism, cholesterol homeostasis, xenobiotic metabolism, and bile acid metabolism were among the top enriched pathways (Fig. 1I). This finding was corroborated by KEGG pathway analyses, which similarly demonstrated top pathways linked to metabolism such as oxidative phosphorylation, glycolysis / gluconeogenesis, retinol, fructose / mannose, pyruvate, drug metabolism, and bile secretion (Fig. 1J). Enrichment of HALLMARK and KEGG signatures in IR-sensitive PDCLs compared to IR-resistant cells are shown in Fig. S3B-C and Table S5. Collectively, these data point to prominently enriched metabolism signatures in IR-resistant PDCLs, suggesting a potential reliance on metabolic pathways.

### Pharmacological screening identifies metabolic pathways as therapeutic targets in IR-resistant PDCLs

**Fig. 2:**
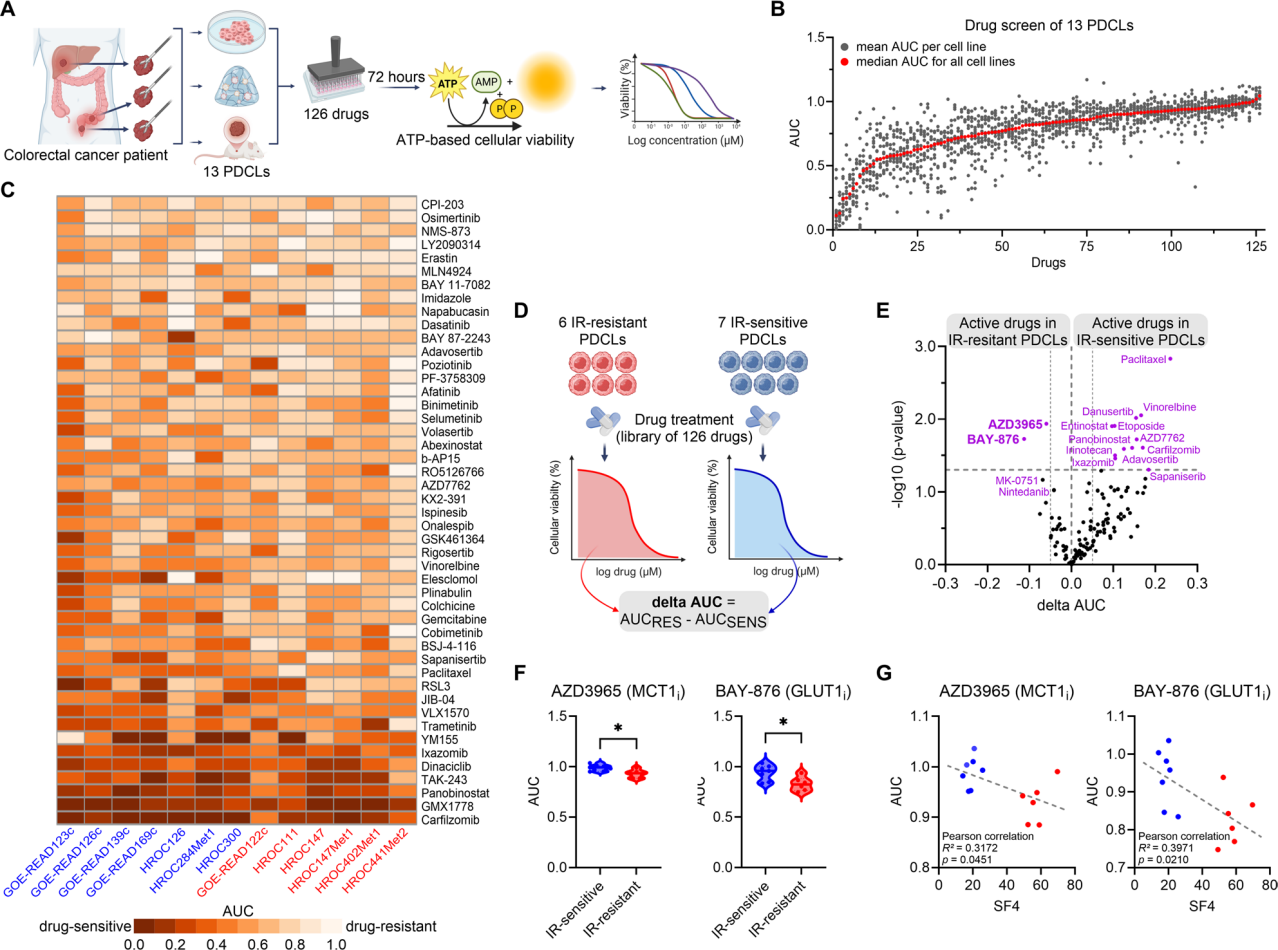
Identification of active compounds in (chemo)irradiation-resistant patient-derived cell lines by drug screening. **A** Schematic illustration of the drug screen workflow, comprising 126 compounds tested across 13 patient-derived cell lines (PDCLs). **B** Distribution of area under the curve (AUC) values across all drug-cell line combinations. **C** Heatmap of drug responses with meanAUC < 0.75 across the panel. Blue: (chemo)irradiation (IR)-sensitive PDCLs; red: IR-resistant PDCLs. **D** Schematic illustration of the deltaAUC calculation to identify drugs preferentially active in IR-resistant cell lines. **E** Volcano plot of deltaAUC values. **F** meanAUC values of the MCT1 inhibitor AZD3965 (left) and the GLUT1 inhibitor BAY-876 (right) in PDCLs. Blue: IR-sensitive PDCLs; red: IR-resistant PDCLs. Student’s ttest: *p < 0.05. **G** Pearson correlation between AUCs of AZD3965 and survival fractions at 4 Gy (SF4; left), and AUCs of BAY-876 and SF4 (right). Blue: IR-sensitive PDCLs; red: IR-resistant PDCLs. PDCL, patient-derived cell line; AUC, area under the curve; IR, (chemo)irradiation; AUC_RES_, meanAUC in IR-resistant PDCLs; AUC_SENS_, meanAUC in IR-sensitive PDCLs; SF4, survival fraction at 4 Gy

To identify radiosensitizing targets, we performed a drug screening experiment (Fig. 2A) encompassing 126 clinically relevant compounds. The drug library was composed of chemotherapies, transferase inhibitors, and agents targeting cellular signaling cascades, pathways of cell death, DNA damage, cell cycle, proteasome, epigenetics, and metabolism (Fig. S4A). Optimal seeding densities (1,000–10,000 cells/well) were established for all PDCLs by assessing cellular viability at 96 h (Fig. S5). Due to its slow proliferation and minimal viability increase across all densities, the HROC389Met2 cell line was excluded from subsequent drug screening (Fig. S5).

Figure 2B summarizes the AUC values, calculated as the mean of two technical replicates per drug, while Fig. 2C and Fig. S4B show a heatmap of drug responses (AUC < 0.75, darker red = lower AUC = indicating strong efficacy). Methodological robustness was corroborated by repeating the screen for one cell line (HROC147) after a four-month interval, yielding highly concordant AUC values (Pearson: *R²* = 0.9075, *p* < 0.001; Spearman: *R* = 0.893, *p* < 0.0001; Fig. S4C). This confirms the reliability of the screening platform. Given the clinical relevance of 5-FU in CRC treatment, this substance was included into the drug screen. Notably, most PDCLs demonstrated resistance to 5-FU (Fig. S4D-E) in this approach, consistent with long-term irradiation/CRT-based CFA results (Fig. S2A, D) and ATP-based cellular viability assays (Fig. 1F), which also showed no enhanced radiosensitizing effect when 5-FU was administered as a radiosensitizer compared to irradiation alone.

To identify compounds selectively active in IR-resistant PDCLs, deltaAUC values (mean AUC IR-resistant group *minus* mean AUC IR-sensitive group) were computed (Fig. 2D). A deltaAUC larger than ± 0.05 was considered to be differentially active between both groups. Strikingly, from 126 compounds, 58 compounds were found more effective in IR-sensitive PDCLs (deltaAUC > 0.05), and only five compounds (7.9%) showing more effectivity in IR-resistant PDCLs (deltaAUC < -0.05; Fig. 2E), meaning that IR-resistant PDCLs exhibited a broad cross-resistance for irradiation, CRT, and for drug treatment. Furthermore, 13 drugs were significantly more active in IR-sensitive PDCLs (Fig. 2E, upper right quadrant). In IR-resistant PDCLs, a trend toward selective activity for the γ-secretase inhibitor MK-0752 (*p* = 0.0687; Fig. S4F) and the broad kinase inhibitor Nintedanib (*p* = 0.0948; Fig. S4G) was observed, suggesting these pathways as potential therapeutic vulnerabilities. Crucially, the only two inhibitors showing significant selective efficacy in IR-resistant PDCLs were the MCT1 inhibitor AZD3965 (*p* = 0.011; Fig. 2F, left panel), and the GLUT1 inhibitor BAY-876 (*p* = 0.0189; Fig. 2F, right panel). AUC values for both compounds inversely correlated with SF4 (AZD3965: *R²* = 0.3172, *p* = 0.0451, left panel; BAY-876: *R*² = 0.3971, *p* = 0.021; Fig. 2G, right panel), confirming their capacity to reduce cellular viability in IR-resistant PDCLs. These findings pinpoint MCT1 and GLUT1 as key metabolism-associated vulnerabilities selectively targeting IR-resistant colorectal cancer cells, highlighting their potential as therapeutic strategy to overcome CRT resistance.

### Metabolic gene signatures correlate with IR-resistance in preclinical and clinical rectal cancer cohorts

**Fig. 3:**
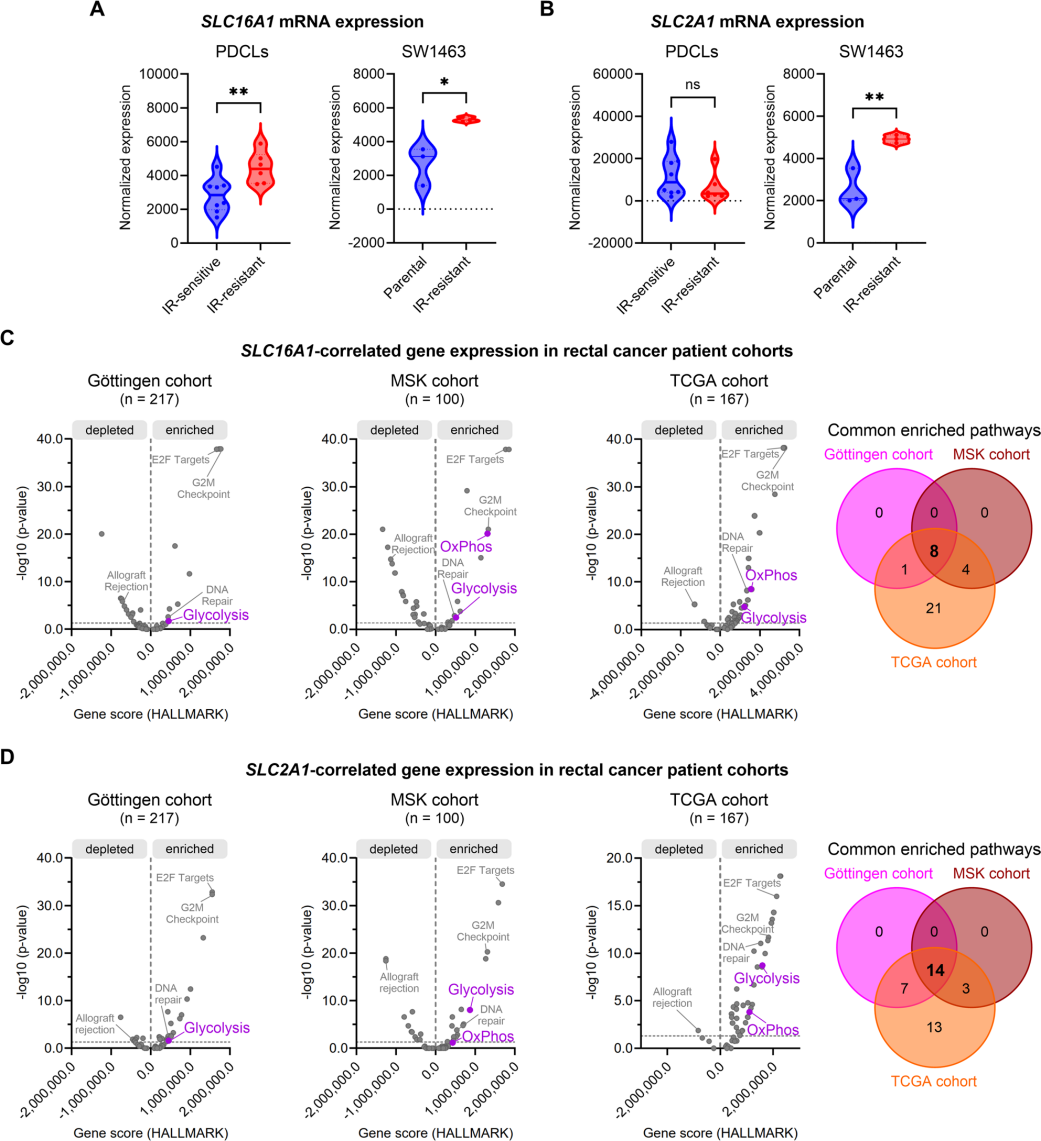
MCT1- and GLUT1-associated pathway activity in cell line models and in rectal cancer patient cohorts. **A** Normalized mRNA expression of *SLC16A1* (encoding MCT1) and **B** of *SLC2A1* (encoding GLUT1) in (chemo)irradiation (IR)-sensitive vs. IR-resistant PDCLs, and in parental SW1463_PAR vs. irradiation-resistant SW1463_RES. Student’s ttest: ns = not significant; *p < 0.05; **p < 0.01. **C** Gene set enrichment analyses (HALLMARK) of *SLC16A1*-correlated gene expression in three rectal cancer patient cohorts (left): Göttingen cohort, MSK cohort, and TCGA cohort. Venn diagram of common enriched pathways (right). **D** Gene set enrichment analyses (HALLMARK) of *SLC2A1*-correlated gene expression across three rectal cancer patient cohorts (left): Göttingen cohort, MSK cohort, and TCGA cohort. Venn diagram of common enriched pathways (right). *SLC16A1*, solute carrier family 16 member 1; *SLC2A1*, solute carrier family 2 member 1; IR, (chemo)irradiation

MCT1 and GLUT1 are both essential for cellular energy metabolism, with MCT1 serving as a transporter for lactate, pyruvate, and ketone bodies, and GLUT1 mediating the cellular uptake of glucose. *SLC16A1* encoding MCT1 and *SLC2A1* encoding GLUT1 are the molecular targets of AZD3965 and BAY-876, respectively. RNA-seq analysis revealed significantly elevated *SLC16A1* expression in IR-resistant PDCLs (*p* = 0.0085; Fig. 3A, left panel), while *SLC2A1* showed no differential expression in PDCLs (*p* = 0.2861; Fig. 3B, left panel). In our isogenic IR-resistant SW1463 model [20], both genes were markedly up-regulated in IR-resistant SW1463_RES versus parental SW1463_PAR (*SLC16A1*: *p* = 0.0167; Fig. 3A, right panel; *SLC2A1*: *p* = 0.0096; Fig. 3B, right panel; Table S8).

To extend this finding to the clinical setting, we correlated *SLC16A1* and *SLC2A1* gene expression with transcriptomic data from three rectal cancer cohorts: the Göttingen [40], MSK [45], and TCGA [46] cohorts. GSEA of the HALLMARK gene sets revealed eight consensus pathways that were consistently enriched among *SLC16A1*-correlated genes across all cohorts (Fig. 3C; Table S9), including glycolysis, DNA repair, E2F targets, G2M checkpoint, unfolded protein response, mTORC1 signaling, and MYC targets (V1/V2). Similarly, *SLC2A1* correlation revealed 14 overlapping enriched pathways (Fig. 3D; Table S9), encompassing glycolysis, DNA repair, E2F targets, G2M checkpoint, cholesterol homeostasis, unfolded protein response, estrogen response early, p53 pathway, UV response up, hypoxia, mitotic spindle, MTORC1 signaling, and MYC targets (V1/V2). Oxidative phosphorylation was found to be enriched for *SLC16A1*- and *SLC2A1-*correlated genes in MSK and TCGA cohorts (Fig. 3C-D). These findings align with oxidative phosphorylation and glycolysis enrichment in IR-resistant PDCLs (Fig. 1I-J), underscoring metabolic reprogramming as a relevant feature of IR resistance.

**Fig. 4:**
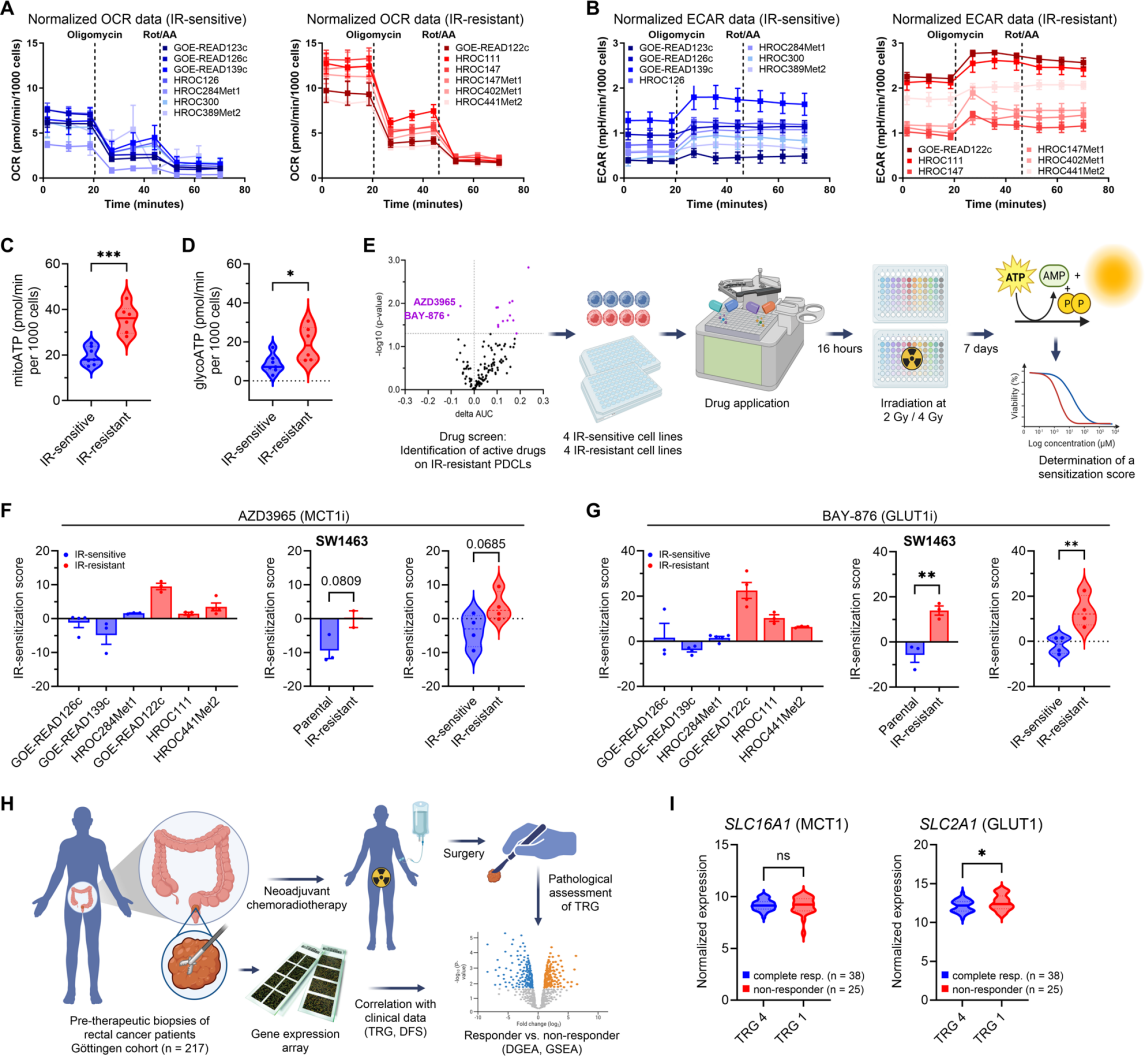
Validation of cellular respiration and glycolysis as potential targets for radiosensitization in preclinical models and in rectal cancer patients treated with chemoradiotherapy. **A-B** Normalized oxygen consumption rates (OCR, **A**) and extracellular acidification rates (ECAR, **B**) measured by Seahorse^®^ XF Analyzer in (chemo)irradiation (IR)-sensitive (blue; left) vs. IR-resistant (red; right) patient-derived cell lines (PDCLs). **C-D** Normalized mitochondrial (**C**) and glycolytic (**D**) ATP production in IR-sensitive (blue) and IR-resistant (red) PDCLs. Student’s ttest: *p < 0.05; ***p < 0.001. **E** Workflow of the robot-assisted drug screening under irradiation and control conditions. **F-G** Irradiation-sensitization scores (ISS) for AZD3965 (**F**) and BAY-876 (**G**) in three IR-sensitive (blue) and three IR-resistant (red) PDCLs as well as in IR-sensitive parental SW1463 (blue) and IR-resistant SW1463_RES cells (red). Student’s ttest: **p < 0.01. **H** Schematic workflow from biopsy to transcriptomics, rectal cancer patient data generation, and correlation with clinical data. **I** Normalized expression of *SLC16A1* (left) and *SLC2A1* (right) in chemoradiotherapy-resistant tumors (TRG 1; red) vs. complete responders (TRG 4; blue) Student’s ttest: ns = not significant; *p < 0.05. OCR, oxygen consumption rate; ECAR, extracellular acidification rate; mitoATP, mitochondrial ATP production; glycoATP, glycolytic ATP production; IR, (chemo)irradiation; PDCL, patient-derived cell line; ISS, irradiation-sensitization score; TRG, tumor regression grade

To functionally validate metabolic alterations, we analyzed cellular energy metabolism by measurement of oxygen consumption rates (OCR), extracellular acidification rates (ECAR), and ATP production rates using seven IR-sensitive (GOE-READ123c, GOE-READ126c, GOE-READ139c, HROC126, HROC284Met1, HROC300, HROC389Met2) and six IR-resistant PDCLs (GOE-READ122c, HROC111, HROC147, HROC147Met1, HROC402Met1, HROC441Met2). Metabolically, both groups exhibited prominent differences, as IR-resistant PDCLs exhibited elevated basal OCR (Fig. 4A, right panel) and ECAR (Fig. 4B, right panel) when compared to IR-sensitive PDCLs (Fig. 4A-B, left panels), indicating enhanced oxidative and glycolytic metabolism. Consequently, mitochondrial ATP production (mitoATP; *p* = 0.0001; Fig. 4C) and glycolytic ATP production (glycoATP; *p* = 0.0182; Fig. 4D) were significantly higher in IR-resistant PDCLs, functionally validating the gene expression and pathway analyses.

### Metabolic inhibition re-sensitizes IR-resistant cells to radiotherapy

Two metabolism-targeting inhibitors identified by drug screening demonstrated significant selective activity against metabolically active IR-resistant PDCLs, i.e., AZD3965 and BAY-876 (Fig. 2E). To determine the effectiveness of these compounds to re-sensitize IR-resistant PDCLs, dose-response assays (-/+ IR) were performed using a robot-assisted liquid handling system (Fig. 4E). Dose-response curves without irradiation for AZD3965 (Fig. S6A-B) and BAY-876 (Fig. S6F-G) were generated for eight cell lines (six PDCLs and SW1463_PAR/SW1463_RES). In contrast to BAY-876 (Fig. S6F-G), AZD3965 did not induce a substantial reduction in cellular viability (Fig. S6A-B). Therefore, to assess the functional efficacy of AZD3965 as an MCT1 inhibitor, extracellular lactate levels were measured using the Lactate-Glo™ assay. As shown in Figure S6C, treatment with 1 µM or 10 µM AZD3965 resulted in a significant reduction of the lactate concentration in the culture medium across all tested PDCLs. This indicates inhibition of MCT1-mediated lactate export by AZD3965.

An irradiation-sensitization score (ISS; positive = radiosensitization, negative = enhanced IR-resistance) was derived in analogy to the Highest Single Agent (HSA) model [28]. AZD3965 radiosensitized IR-resistant PDCLs (GOE-READ122c: ISS = 9.52; HROC111: ISS = 1.48; HROC441Met2: ISS = 3.48; Fig. 4F, left panel), while IR-sensitive lines showed neutral or even resistance-inducing effects (GOE-READ126c: ISS = − 1.20; GOE-READ139c: ISS = − 4.87; HROC284Met1: ISS = 1.58; Fig. 4F, left panel). Although SW1463_RES was unaffected (Fig. 4F, middle panel), analysis of all cellular models trended toward radiosensitization in IR-resistant cells (*p* = 0.0685; Fig. 4F, right panel). To exclude compensatory upregulation of other lactate transporters (e.g., MCT2, MCT3, or MCT4), which might have attenuated the radiosensitizing effect of AZD3965-mediated MCT1 inhibition, we investigated mRNA expression. First, we investigated the expression levels of *SLC16A1* (MCT1), *SLC16A7* (MCT2), *SLC16A8* (MCT3), and *SLC16A3* (MCT4) in our PDCL panel. RNA-seq analysis had identified only *SLC16A1* (MCT1) and *SLC16A3* (MCT4) as sufficiently expressed in the PDCLs (Fig. S6D, Fig. 3A). In addition, treatment with 10 µM AZD3965 did not induce deregulation of either *SLC16A1* or *SLC16A3* in any of the six tested PDCLs (Fig. S6E), suggesting that inhibition of MCT1 is not counteracted by compensatory expression changes in other lactate transporters.

BAY-876 demonstrated stronger effects in all IR-resistant cell models, with GOE-READ122c showing maximal sensitization to IR (ISS = 22.49), while IR-sensitive cell lines showed no effect on IR or even an increase in IR-resistance (Fig. 4G, left and middle panel). Taken together, IR-resistant PDCLs and SW1463_RES exhibited significantly enhanced radiosensitivity versus IR-sensitive counterparts upon blockade of glucose entry (*p* = 0.0089; Fig. 4G, right panel).

### Expression of SLC2A1 correlates with treatment response in the clinical setting

Given that *SLC16A1* (MCT1) and *SLC2A1* (GLUT1) are critical regulators of metabolism, and that their pharmacological inhibition re-sensitizes IR-resistant colorectal cancer cells to irradiation, we investigated the potential clinical relevance in therapy resistance among rectal cancer patients.

Gene expression data from the Göttingen cohort were generated from 217 pre-therapeutic biopsies (Fig. 4H) [40], which can be correlated with the respective histopathological response rates after neoadjuvant therapy. For all surgical specimens, TRG was determined [48]: A TRG of 4 refers to a complete histopathological tumor regression, whereas a TRG of 1 indicates a resistant tumor. In the Göttingen cohort, there were 25 patients with a TRG 1, and 38 patients with a TRG of 4. Importantly, while *SLC16A1* expression did not differ between both groups (*p* = 0.7152; Fig. 4I, left panel), non-responsive tumors (TRG 1) showed significantly higher *SLC2A1* levels than tumors with a complete CRT-response (TRG 4; *p* = 0.0408; Fig. 4I, right panel). Therefore, we conclude that metabolic dependency, specifically a reliance on glycolysis, is associated with radioresistance, presenting a viable strategy for patient stratification and targeted intervention.

## Discussion

Response of LARC to neoadjuvant treatment strategies is restricted by intrinsic therapy resistance. This is reflected by pathological complete response (pCR) rates, i.e., full regression of both the primary tumor as well as the locoregional lymph nodes after neoadjuvant treatment, in only 20–30% of patients [52–56]. As extensively shown, pCR represents a surrogate endpoint for improved survival [13, 52, 54, 56]. Importantly, up to 40% of patients experience a clinical complete response (cCR) after (total) neoadjuvant treatment, and may therefore be spared from radical surgery (organ-preserving watch-and-wait strategy) [1, 7–12]. The inability to elicit a complete response in a higher fraction of patients defines a therapeutic gap that demands new biological insights and intervention strategies.

Here, we employed an integrative and mechanistic approach to dissect irradiation and CRT response in a panel of 14 primary patient-derived colorectal cancer 2D cell lines, isolated from primary tumors, PDX- or PDO-model systems. Although we did not compare the molecular alteration of the primary tumors with those of our 2D models, and although different selection pressures during the isolation process may influence these alterations, our reverse-translational approach demonstrates that metabolic dependencies are relevant in the clinical patient cohorts investigated, thereby underscoring the validity of our model platform in representing a relevant spectrum of RC resistance phenotypes.

We establish that metabolic reprogramming is a relevant driver of irradiation and CRT resistance and demonstrate the therapeutic potential of targeting this vulnerability. We identified metabolic pathways associated with irradiation and CRT resistance in cellular models and primary cancers undergoing neoadjuvant therapy and validated elevated glycolysis and oxidative phosphorylation using metabolic read-out systems. Our data are consistent with a recent systematic integrative meta-analysis of RC omics datasets, which identified amino-acid metabolism, glutathione-ROS pathways, and glycolysis as the metabolic programs most strongly associated with resistance to neoadjuvant treatment [57]. Importantly, the majority of our IR-resistant models (5/6) were derived from patients who did not receive neoadjuvant irradiation or CRT therapy prior to tumor resection (three untreated, two chemotherapy-only), indicating that metabolic reprogramming may confer a primary resistance pathway rather than being solely a consequence of treatment exposure. This observation aligns with clinical evidence that metabolic vulnerabilities, such as elevated GLUT1 expression, predict poor response to neoadjuvant therapy regardless of prior treatment history [58]. Although our cell line panel captures a degree of genetic heterogeneity, establishing associations between specific genetic events and metabolic dependencies requires the analysis of larger cell line panels and patient cohorts.

Drug-screening of 126 clinically relevant substances showed that only two metabolism-targeting compounds, the MCT1 inhibitor AZD3965 and the GLUT1 inhibitor BAY-876, displayed increased activity in the resistant PDCLs, indicating that metabolic dependency represents a targetable vulnerability. Importantly, BAY-876 markedly induced radiosensitization, and CRT-resistant rectal cancers exhibited significantly higher GLUT1 mRNA expression than pathological complete responders, supporting a relevant role for glycolysis in mediating resistance. Consistently, GLUT1 expression assessed by immunohistochemistry was associated with a reduced response to neoadjuvant therapy and shorter DFS in an independent study [58]. The connection between heightened glycolysis and radioresistance is well documented. Beyond other effects, elevated glycolysis may also contribute to cross-resistance against chemotherapeutic agents commonly used in neoadjuvant regimens for RC. Metabolic reprogramming toward heightened glycolytic flux evidenced by elevated GLUT1 expression and higher activity of glycolysis in CRT-resistant tumors can simultaneously attenuates oxidative stress responses critical for both radiation- and chemotherapy-induced DNA damage. Specifically, increased glycolytic flux may expand the pentose-phosphate pathway to fuel nucleotide synthesis for DNA repair, while elevating NADPH and glutathione levels to neutralize reactive oxygen species (ROS) generated by both ionizing radiation and cytotoxic chemotherapies [59–61]. This shared metabolic mechanism can explain why targeting glycolysis with GLUT1 inhibition (e.g., BAY-876) not only radiosensitizes but also potentiates chemosensitivity in resistant models [62]. Consequently, metabolic dependencies represent a unified therapeutic strategy to overcome dual resistance to chemoradiotherapy, aligning with the observed clinical correlation between high GLUT1 expression and reduced pathological complete response rates across CRT regimens [58]. However, the causal relationship between enhanced glycolysis, GLUT1 activity, and cross-resistance to chemoradiotherapy requires further experimental validation.

Because glycolysis contributes substantially to tumor growth and therapeutic resistance, a variety of glycolytic inhibitors have been designed and evaluated, though none have progressed to regulatory approval to date [63]. While GLUT1 inhibitors are currently confined to preclinical development, the MCT1 inhibitor AZD3965 has progressed into clinical trials, where it has shown a manageable safety profile [64]. Furthermore, inhibition of PFKFB3 (6-phosphofructo-2-kinase/fructose-2,6-bisphosphatase-3), for which no compounds were represented in our screening library, remains an alternative strategy to disrupt glycolysis. The PFKFB3 inhibitor PFK-158 entered a phase I clinical study [65] and the PFKFB3 inhibitor KAN0438757 has a clear therapeutic window in preclinical model systems [66]. In line with the observations, the PFKFB3 inhibitor 2E-3-(3-pyridinyl)-1-(4-pyridinyl)-2-propen-1-one (3PO) was recently reported to enhance the radiosensitivity of CRC cells in vitro [67], reinforcing the significance of our findings.

### Limitations of our study

The limited availability of agents capable of directly targeting glycolysis constrains the translational potential of our findings, but it also highlights the need to develop glycolysis inhibitors with a defined therapeutic window, including testing of agents that modulate glycolysis indirectly. As another opportunity, drug repurposing represents a promising strategy to accelerate clinical translation, as several commonly used drugs (e.g., NSAIDs, metformin) have been shown to indirectly modulate glycolytic pathways through HIF-1α suppression [63]. Although a direct contribution of the additional metabolic pathways identified in our study to CRT sensitization remains unclear, our findings highlight the importance of investigating and ultimately exploiting these pathways in future translational research.

## Conclusion

Given its key role in promoting radioresistance in RC, monitoring the tumor’s glycolytic activity may provide a basis for stratifying patients who could benefit from more intensive, metabolism-targeted therapeutic approaches.

## Supplementary Information

Supplementary Material 1.

Supplementary Material 2.

Supplementary Material 3.

Supplementary Material 4.

Supplementary Material 5.

Supplementary Material 6.

Supplementary Material 7.

Supplementary Material 8.

Supplementary Material 9.

Supplementary Material 10.

## Data Availability

Source data are available upon request. Data from the RNA-seq of the 14 PDCLs can be accessed via Table S4. Data from SW1463 RNA-seq can be accessed via Table S8. Previously published microarray data of the Göttingen rectal cancer patient cohort can be accessed via GSE87211 [40].
