# Supplementary material for: Targeting metabolic dependencies to reverse chemoradiotherapy resistance in colorectal cancer
Source: J Exp Clin Cancer Res. 2026 Jun 23;45:143. doi: 10.1186/s13046-026-03755-x (PMC13292332; doi:10.1186/s13046-026-03755-x)
Supplement: Supplementary file 4 — Supplementary Material 4. [file 13046_2026_3755_MOESM4_ESM.docx]

**Supplementary Table S3. Experimental conditions**

**Supplementary Table 3a**. Live-cell imaging and irradiation experiments

| **Cell line** | **Cell number** | **Irradiation dose** | **Incubation time** | **Incucyte® segmentation mode** | **Segmentation adjustance** | **Hole fill (µm²)** | **Adjusted size (pixel)** | **Area min (µm²)** | **Eccentricity max.** |
| --- | --- | --- | --- | --- | --- | --- | --- | --- | --- |
| GOE-READ122c | 4,000 | 4 Gy | 240 h | AI Confluence | - | 4,000 | 0 | 250 | 0 |
| GOE-READ123c | 3,000 | 4 Gy | 240 h | AI Confluence | - | 4,000 | -1 | 100 | 0 |
| GOE-READ126c | 3,000 | 4 Gy | 240 h | AI Confluence | - | 0 | 0 | 250 | 0 |
| GOE-READ139c | 750 | 4 Gy | 240 h | AI Confluence | - | 1.00E+05 | -1 | 0 | 0 |
| GOE-READ169c | 1,000 | 4 Gy | 240 h | AI Confluence | - | 1.00E+05 | 0 | 0 | 0 |
| HROC111 | 2,000 | 4 Gy | 240 h | AI Confluence | - | 2.00E+05 | 0 | 500 | 0 |
| HROC126 | 2,000 | 4 Gy | 240 h | AI Confluence | - | 1000 | -1 | 100 | 0 |
| HROC147 | 1,500 | 4 Gy | 240 h | AI Confluence | - | 1.00E+05 | 0 | 0 | 0.97 |
| HROC147Met1 | 1,000 | 4 Gy | 240 h | AI Confluence | - | 1000 | 0 | 250 | 0 |
| HROC284Met1 | 750 | 4 Gy | 240 h | AI Confluence | - | 8700 | 0 | 250 | 0 |
| HROC300 | 5,000 | 4 Gy | 240 h | AI Confluence | - | 1.00E+04 | 0 | 150 | 0 |
| HROC389Met2 | 6,000 | 4 Gy | 240 h | AI Confluence | - | 1.00E+04 | 0 | 250 | 0 |
| HROC402Met1 | 1,500 | 4 Gy | 240 h | AI Confluence | - | 1.00E+04 | 0 | 250 | 0 |
| HROC441Met2 | 3,000 | 4 Gy | 240 h | AI Confluence | - | 1.00E+05 | 0 | 250 | 0 |

**Supplementary Table 3b**. Chemoirradiation experiments

| **Cell line** | **Cell number** | **Chemotherapy (CT) dose** | **CT incubation time prior to irradiation** | **Irradiation dose** | **Incubation time** |
| --- | --- | --- | --- | --- | --- |
| GOE-READ122c | 5,000 | 3 µM 5-FU + 1 µM Oxaliplatin | 16 h | 4 Gy | 240 h |
| GOE-READ123c | 4,000 | 3 µM 5-FU + 1 µM Oxaliplatin | 16 h | 4 Gy | 240 h |
| GOE-READ126c | 3,000 | 3 µM 5-FU + 1 µM Oxaliplatin | 16 h | 4 Gy | 240 h |
| GOE-READ139c | 750 | 3 µM 5-FU + 1 µM Oxaliplatin | 16 h | 4 Gy | 240 h |
| GOE-READ169c | 1,000 | 3 µM 5-FU + 1 µM Oxaliplatin | 16 h | 4 Gy | 240 h |
| HROC111 | 1,500 | 3 µM 5-FU + 1 µM Oxaliplatin | 16 h | 4 Gy | 240 h |
| HROC126 | 2,000 | 3 µM 5-FU + 1 µM Oxaliplatin | 16 h | 4 Gy | 240 h |
| HROC147 | 3,000 | 3 µM 5-FU + 1 µM Oxaliplatin | 16 h | 4 Gy | 240 h |
| HROC147Met1 | 3,000 | 3 µM 5-FU + 1 µM Oxaliplatin | 16 h | 4 Gy | 240 h |
| HROC284Met1 | 750 | 3 µM 5-FU + 1 µM Oxaliplatin | 16 h | 4 Gy | 240 h |
| HROC300 | 4,000 | 3 µM 5-FU + 1 µM Oxaliplatin | 16 h | 4 Gy | 240 h |
| HROC389Met2 | 6,000 | 3 µM 5-FU + 1 µM Oxaliplatin | 16 h | 4 Gy | 240 h |
| HROC402Met1 | 4,000 | 3 µM 5-FU + 1 µM Oxaliplatin | 16 h | 4 Gy | 240 h |
| HROC441Met2 | 3,000 | 3 µM 5-FU + 1 µM Oxaliplatin | 16 h | 4 Gy | 240 h |

**Supplementary Table 3c**. Colony formation assay (CFA)

| **Cell line** | **Treatment** | **Cell number 0 Gy** | **Cell number 0.5 Gy** | **Cell number 1 Gy** | **Cell number 2 Gy** | **Cell number 4 Gy** | **Cell number 6 Gy** | **CFA growth period (days)** |
| --- | --- | --- | --- | --- | --- | --- | --- | --- |
| GOE-READ139c | Irradiation only | 1,500 | 1,500 | 1,500 | 3,000 | 9,000 | 12,000 | 10 |
|  | Irradiation + 3 µM 5-FU | 1,500 | 1,500 | 1,500 | 3,000 | 9,000 | 12,000 | 10 |
| GOE-READ169c | Irradiation only | 4,500 | 4,500 | 4,500 | 9,000 | 13,500 | 18,000 | 10 |
|  | Irradiation + 3 µM 5-FU | 4,500 | 4,500 | 4,500 | 9,000 | 13,500 | 18,000 | 10 |
| HROC126 | Irradiation only | 4,000 | 4,000 | 4,000 | 8,000 | 12,000 | 16,000 | 17 |
|  | Irradiation + 3 µM 5-FU | 4,000 | 4,000 | 4,000 | 8,000 | 12,000 | 16,000 | 17 |
| HROC147 | Irradiation only | 2,000 | 2,000 | 2,000 | 2,000 | 4,000 | 6,000 | 16 |
|  | Irradiation + 3 µM 5-FU | 2,000 | 2,000 | 2,000 | 2,000 | 4,000 | 6,000 | 16 |
| HROC147Met1 | Irradiation only | 2,000 | 2,000 | 2,000 | 2,000 | 4,000 | 6,000 | 14 |
|  | Irradiation + 3 µM 5-FU | 2,000 | 2,000 | 2,000 | 2,000 | 4,000 | 6,000 | 14 |
| HROC284Met1 | Irradiation only | 1,000 | 1,000 | 1,000 | 1,000 | 2,000 | 3,000 | 11 |
|  | Irradiation + 3 µM 5-FU | 1,000 | 1,000 | 1,000 | 1,000 | 2,000 | 3,000 | 11 |
| HROC300 | Irradiation only | 2,000 | 2,000 | 2,000 | 2,000 | 4,000 | 6,000 | 22 |
|  | Irradiation + 3 µM 5-FU | 2,000 | 2,000 | 2,000 | 2,000 | 4,000 | 6,000 | 22 |

**Supplementary Table S3d**. Drug screening

| **Cell line** | **Cell number** | **Treatment** | **Incubation time** |
| --- | --- | --- | --- |
| GOE-READ122c | 6,000 | 126 drugs (Supp. Fig. S4) | 72 h |
| GOE-READ123c | 3,000 | 126 drugs (Supp. Fig. S4) | 72 h |
| GOE-READ126c | 3,000 | 126 drugs (Supp. Fig. S4) | 72 h |
| GOE-READ139c | 1,500 | 126 drugs (Supp. Fig. S4) | 72 h |
| GOE-READ169c | 2,000 | 126 drugs (Supp. Fig. S4) | 72 h |
| HROC111 | 5,000 | 126 drugs (Supp. Fig. S4) | 72 h |
| HROC126 | 15,000 | 126 drugs (Supp. Fig. S4) | 72 h |
| HROC147 | 4,000 | 126 drugs (Supp. Fig. S4) | 72 h |
| HROC147Met1 | 8,000 | 126 drugs (Supp. Fig. S4) | 72 h |
| HROC284Met1 | 1,500 | 126 drugs (Supp. Fig. S4) | 72 h |
| HROC300 | 10,000 | 126 drugs (Supp. Fig. S4) | 72 h |
| HROC402Met1 | 5,000 | 126 drugs (Supp. Fig. S4) | 72 h |
| HROC441Met2 | 5,000 | 126 drugs (Supp. Fig. S4) | 72 h |

**Supplementary Table 3e**. Robot-assisted functional validation of targeted inhibitors

| **Cell line** | **Cell number** | **Drug treatment** | **Irradiation doses** | **Incubation time** |
| --- | --- | --- | --- | --- |
| GOE-READ122c | 5,000 | AZD3965 | 0 Gy; 4 Gy | 168 h |
|  | 5,000 | BAY-876 | 0 Gy; 4 Gy | 168 h |
|  | 5,000 | Nintedanib | 0 Gy; 4 Gy | 168 h |
|  | 5,000 | MK0752 | 0 Gy; 4 Gy | 168 h |
| GOE-READ126c | 4,000 | AZD3965 | 0 Gy; 2 Gy | 168 h |
|  | 4,000 | BAY-876 | 0 Gy; 2 Gy | 168 h |
|  | 4,000 | Nintedanib | 0 Gy; 2 Gy | 168 h |
|  | 4,000 | MK0752 | 0 Gy; 2 Gy | 168 h |
| GOE-READ139c | 750 | AZD3965 | 0 Gy; 2 Gy | 168 h |
|  | 750 | BAY-876 | 0 Gy; 2 Gy | 168 h |
|  | 750 | Nintedanib | 0 Gy; 2 Gy | 168 h |
|  | 750 | MK0752 | 0 Gy; 2 Gy | 168 h |
| HROC111 | 2,000 | AZD3965 | 0 Gy; 4 Gy | 168 h |
|  | 2,000 | BAY-876 | 0 Gy; 4 Gy | 168 h |
|  | 2,000 | Nintedanib | 0 Gy; 4 Gy | 168 h |
|  | 2,000 | MK0752 | 0 Gy; 4 Gy | 168 h |
| HROC284Met1 | 750 | AZD3965 | 0 Gy; 2 Gy | 168 h |
|  | 750 | BAY-876 | 0 Gy; 2 Gy | 168 h |
|  | 750 | Nintedanib | 0 Gy; 2 Gy | 168 h |
|  | 750 | MK0752 | 0 Gy; 2 Gy | 168 h |

**Supplementary Table 3e (continued)**. Robot-assisted functional validation of targeted inhibitors

| **Cell line** | **Cell number** | **Drug treatment** | **Irradiation doses** | **Incubation time** |
| --- | --- | --- | --- | --- |
| HROC441Met2 | 4,000 | AZD3965 | 0 Gy; 4 Gy | 168 h |
|  | 4,000 | BAY-876 | 0 Gy; 4 Gy | 168 h |
|  | 4,000 | Nintedanib | 0 Gy; 4 Gy | 168 h |
|  | 4,000 | MK0752 | 0 Gy; 4 Gy | 168 h |
| SW1463_PAR | 3,000 | AZD3965 | 0 Gy; 4 Gy | 168 h |
|  | 3,000 | BAY-876 | 0 Gy; 4 Gy | 168 h |
|  | 3,000 | Nintedanib | 0 Gy; 4 Gy | 168 h |
|  | 3,000 | MK0752 | 0 Gy; 4 Gy | 168 h |
| SW1463_RES | 3,000 | AZD3965 | 0 Gy; 4 Gy | 168 h |
|  | 3,000 | BAY-876 | 0 Gy; 4 Gy | 168 h |
|  | 3,000 | Nintedanib | 0 Gy; 4 Gy | 168 h |
|  | 3,000 | MK0752 | 0 Gy; 4 Gy | 168 h |

**Supplementary Table 3f**. Seahorse^®^ assay

| **Cell line** | **Cell number** | **Treatment #1 / incubation time** | **Treatment #2 / incubation time** | **Total incubation time** |
| --- | --- | --- | --- | --- |
| GOE-READ122c | 30,000 | Oligomycin / 24 min | Rotenone/Antimycin A (Rot/AA) / 24 min | 72 min |
| GOE-READ123c | 20,000 | Oligomycin / 24 min | Rotenone/Antimycin A (Rot/AA) / 24 min | 72 min |
| GOE-READ126c | 30,000 | Oligomycin / 24 min | Rotenone/Antimycin A (Rot/AA) / 24 min | 72 min |
| GOE-READ139c | 6,000 | Oligomycin / 24 min | Rotenone/Antimycin A (Rot/AA) / 24 min | 72 min |
| HROC111 | 7,500 | Oligomycin / 24 min | Rotenone/Antimycin A (Rot/AA) / 24 min | 72 min |
| HROC126 | 20,000 | Oligomycin / 24 min | Rotenone/Antimycin A (Rot/AA) / 24 min | 72 min |
| HROC147 | 20,000 | Oligomycin / 24 min | Rotenone/Antimycin A (Rot/AA) / 24 min | 72 min |
| HROC147Met1 | 30,000 | Oligomycin / 24 min | Rotenone/Antimycin A (Rot/AA) / 24 min | 72 min |
| HROC284Met1 | 7,500 | Oligomycin / 24 min | Rotenone/Antimycin A (Rot/AA) / 24 min | 72 min |
| HROC300 | 10,000 | Oligomycin / 24 min | Rotenone/Antimycin A (Rot/AA) / 24 min | 72 min |
| HROC389Met2 | 15,000 | Oligomycin / 24 min | Rotenone/Antimycin A (Rot/AA) / 24 min | 72 min |
| HROC402Met1 | 30,000 | Oligomycin / 24 min | Rotenone/Antimycin A (Rot/AA) / 24 min | 72 min |
| HROC441Met2 | 7,500 | Oligomycin / 24 min | Rotenone/Antimycin A (Rot/AA) / 24 min | 72 min |

**Supplementary Table 3g**. Lactate-Glo^TM^ assay

| **Cell line** | **Cell number** | **Treatment #1 / incubation time** | **Incubation time** |
| --- | --- | --- | --- |
| GOE-READ122c | 30,000 | DMSO / 1 µM AZD3965 / 10 µM AZD3965 | 72 h |
| GOE-READ126c | 30,000 | DMSO / 1 µM AZD3965 / 10 µM AZD3965 | 72 h |
| GOE-READ139c | 6,000 | DMSO / 1 µM AZD3965 / 10 µM AZD3965 | 72 h |
| HROC111 | 7,500 | DMSO / 1 µM AZD3965 / 10 µM AZD3965 | 72 h |
| HROC284Met1 | 7,500 | DMSO / 1 µM AZD3965 / 10 µM AZD3965 | 72 h |
| HROC441Met2 | 7,500 | DMSO / 1 µM AZD3965 / 10 µM AZD3965 | 72 h |

**Supplementary Table 3h**. Primer sequences

| **Gene** | **Accession Number** | **Primer Sequence** | **Size (bp)** | **Company** |
| --- | --- | --- | --- | --- |
| SLC16A1 | NM_003051 | Forward: GAACAAGCAAACGAGGCAGC  Reverse: CAGGTCAAATCCAAATATCGTT | 136 | IDT |
| SLC16A3 | NM_001206950 | Forward: GGATCGGCTACAGCGACAC  Reverse: CGACGCAAAGAGACCCCC | 137 | IDT |
| HPRT1 | NM_000194.2 | Forward: TGACACTGGCAAAACAATGCA  Reverse: GGTCCTTTTCACCAGCAAGCT | 93 | MWG |
