# Supplementary material for: Targeting metabolic dependencies to reverse chemoradiotherapy resistance in colorectal cancer
Source: J Exp Clin Cancer Res. 2026 Jun 23;45:143. doi: 10.1186/s13046-026-03755-x (PMC13292332; doi:10.1186/s13046-026-03755-x)
Supplement: Supplementary file 7 — Supplementary Material 7. [file 13046_2026_3755_MOESM7_ESM.docx]

**Supplementary Figures and Supplementary Figure Legends**


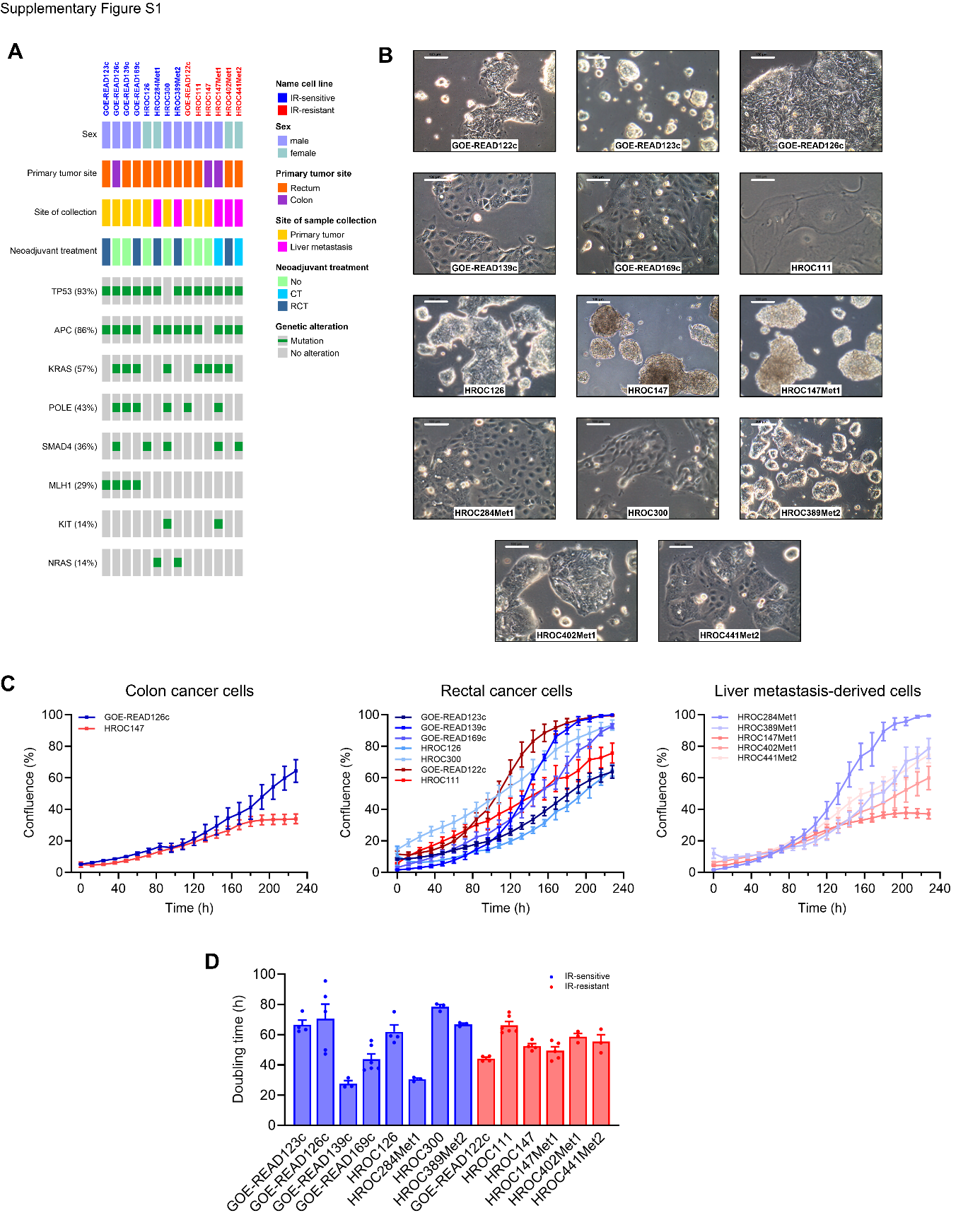


**Figure S1: Characterization of the patient-derived cell line panel.**

**A**) Oncoprint illustration showing genomic alterations across the patient-derived cell line (PDCL) panel, annotated with color code for cell line names, clinical parameters: sex, primary tumor site, site of sample collection, neoadjuvant treatment, and genetic alterations. **B**) Brightfield microscopy images of the 14 distinct PDCLs. Scale bars: 100 µm. **C**) Live-cell imaging-derived growth curves of the PDCL panel, stratified by tissue origin: colon cancer, rectal cancer, and liver metastasis-derived cell lines. **D**) Bar chart summarizing the doubling times of the 14 individual PDCLs, as determined by live-cell imaging. Blue bars: (chemo)irradiation (IR)-sensitive cell lines, red bars: IR-resistant cell lines.

PDCL, patient-derived cell line; IR, (chemo)irradiation.


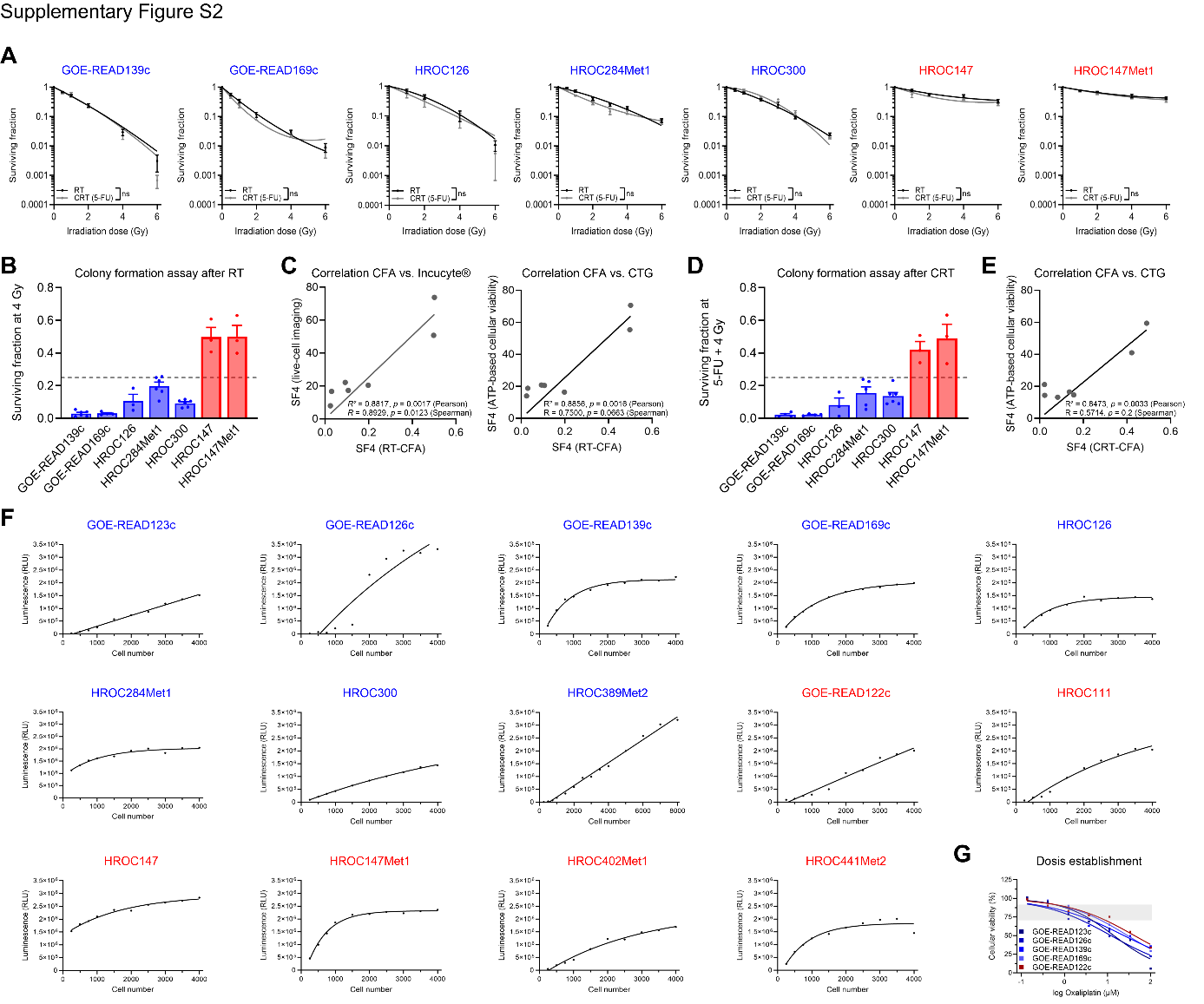


**Figure S2: Irradiation experiments in patient-derived cell lines.**

**A**) Survival fractions following radiotherapy (RT) alone (black) or RT in combination with 3 µM 5-Fluorouracil (5-FU; CRT grey), as assessed by colony formation assays (CFA) in seven PDCLs from the panel. Statistical difference between RT and CRT was tested by ANOVA: ns = not significant. **B**) Bar chart depicting RT-based surviving fractions at 4 Gy (SF4), derived from the data shown in (**A**). **C**) Correlation analyses between different assay platforms for RT response: RT-CFA vs. live-cell imaging (left panel); RT-CFA vs. ATP-based cellular viability (right panel). **D**) Bar chart depicting chemoradiotherapy (CRT)-based SF4, derived from the data shown in (**A**). **E**) Correlation analyses between different assay platforms for CRT response: CRT-CFA vs. ATP-based cellular viability. **F**) Luminescence values measured by ATP-based cellular viability assay 240 hours post-seeding, across a range of increasing cell numbers per well, starting from 250. **G**) ATP-based cellular viability of dose-response curves for Oxaliplatin in five PDCLs with labeling of the IC_20_ region in grey.

PDCL, patient-derived cell line; RT, radiotherapy; 5-FU, 5-Fluorouracil; CFA, colony formation assay; SF4, surviving fraction at 4 Gy; CRT, chemoradiotherapy; RLU, relative light units; IC_20_, inhibitory concentration at 20% viability reduction.


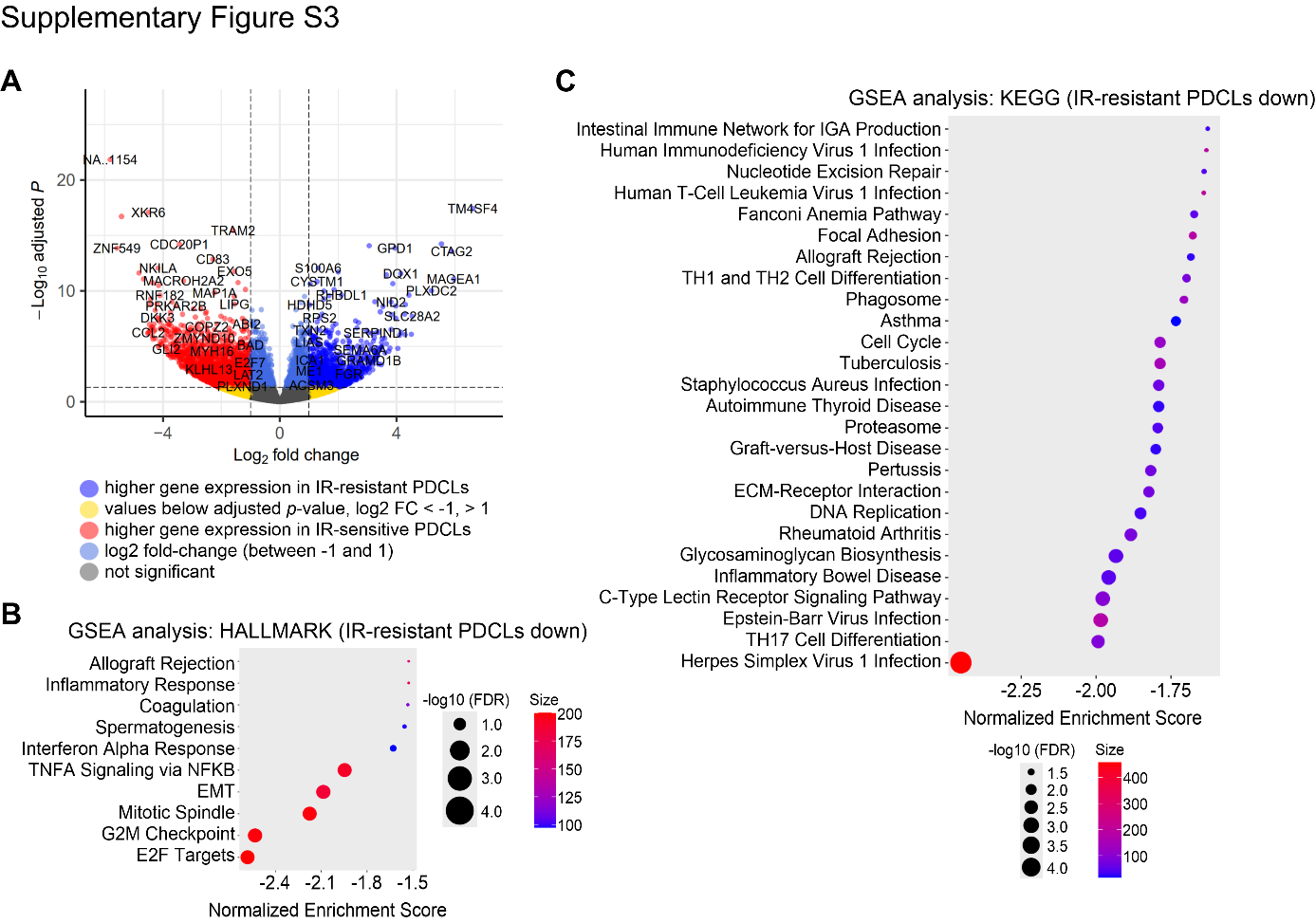


**Figure S3: Differentially expressed genes in (chemo)irradiation-resistant vs. (chemo)irradiation-sensitive patient-derived cell lines.**

**A**) Volcano plot depicting differentially expressed genes between (chemo)irradiation (IR)-resistant and IR-sensitive patient-derived cell lines (PDCLs), based on log2 fold change versus adjusted *p*-values (–log₁₀ adjusted *p*-value). **B-C**) Gene set enrichment analysis (GSEA) comparing IR-sensitive and IR-resistant cell lines using the HALLMARK gene sets (**B**), KEGG gene sets (**C**).

PDCL, patient-derived cell line; IR, (chemo)irradiation; GSEA, gene set enrichment analysis; log2 FC, log2 fold change.


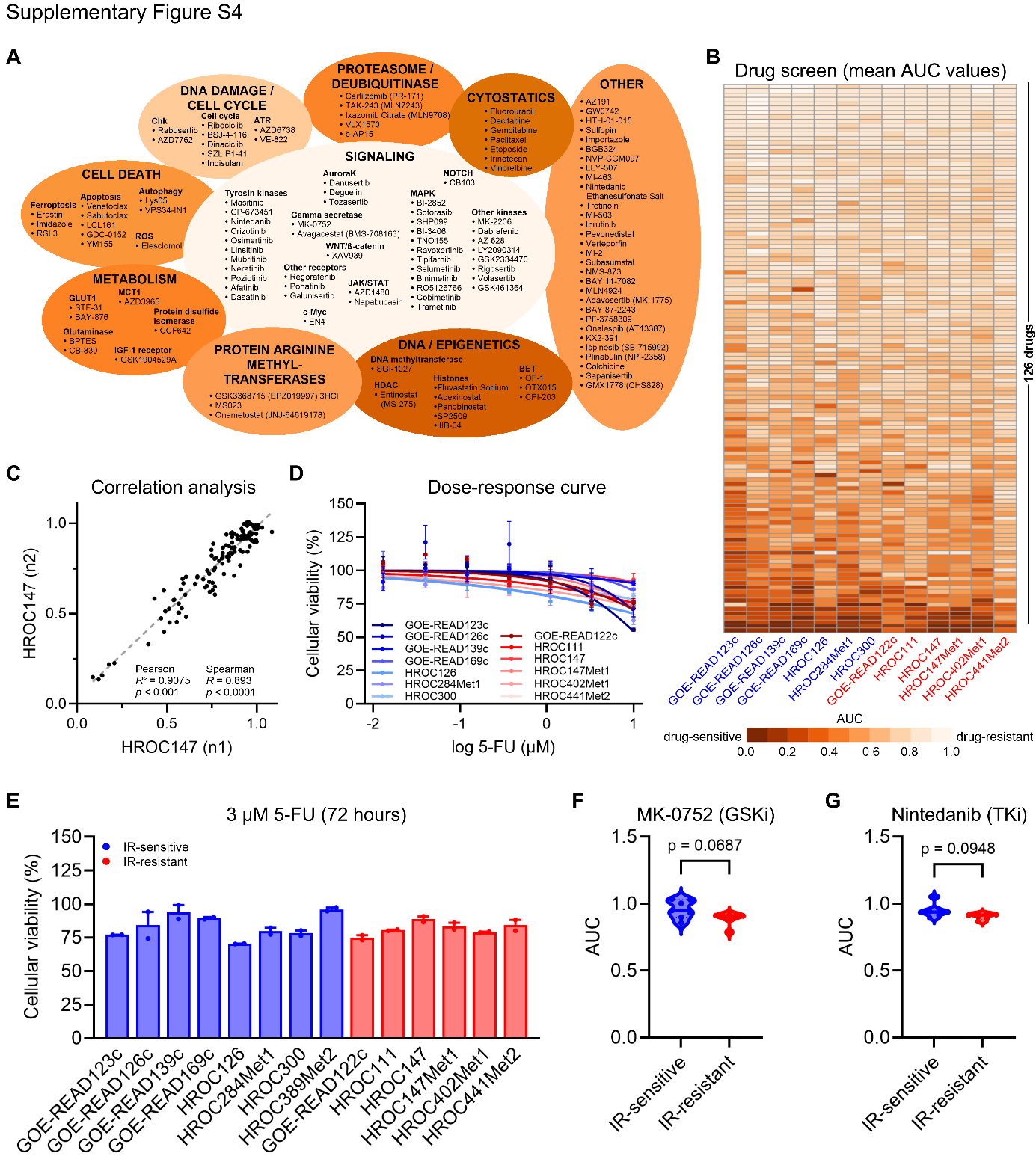


**Figure S4: Drug screening experiments.**

**A**) Overview of the 126 compounds screened, categorized by molecular target. **B**) Heatmap of mean area under the curve (AUC) values across 13 patient-derived cell lines (PDCLs) for 126 drugs. Darker orange indicates lower AUC values (higher drug sensitivity). Blue, (chemo)irradiation (IR)-sensitive PDCLs; red, IR-resistant PDCLs. **C**) Correlation analysis of two biological replicates of the drug screening experiment using the HROC147 cell line for reproducibility evaluation of the drug screen. **D**) Dose-response curves for 5-Fluoruracil (5-FU) across 13 PDCLs (derived from drug screen). **E**) ATP-based cellular viability assay of 13 PDCLs after treatment with 3 µM 5-FU for 72 hours (derived from drug screen). Colors denote IR-sensitive (blue) and IR-resistant (red) cell lines. **F-G**) Violin plots of meanAUC values for MK-0752 (GSK_i_, gamma secretase inhibitor, (**F**)) and Nintedanib (TK_i_, tyrosine kinase inhibitor, (**G**)) in IR-sensitive vs. IR-resistant PDCLs. Significance values were calculated by Student’s ttest.

AUC, area under the curve; PDCL, patient-derived cell line; IR, (chemo)irradiation; 5-FU, 5-Fluorouracil.


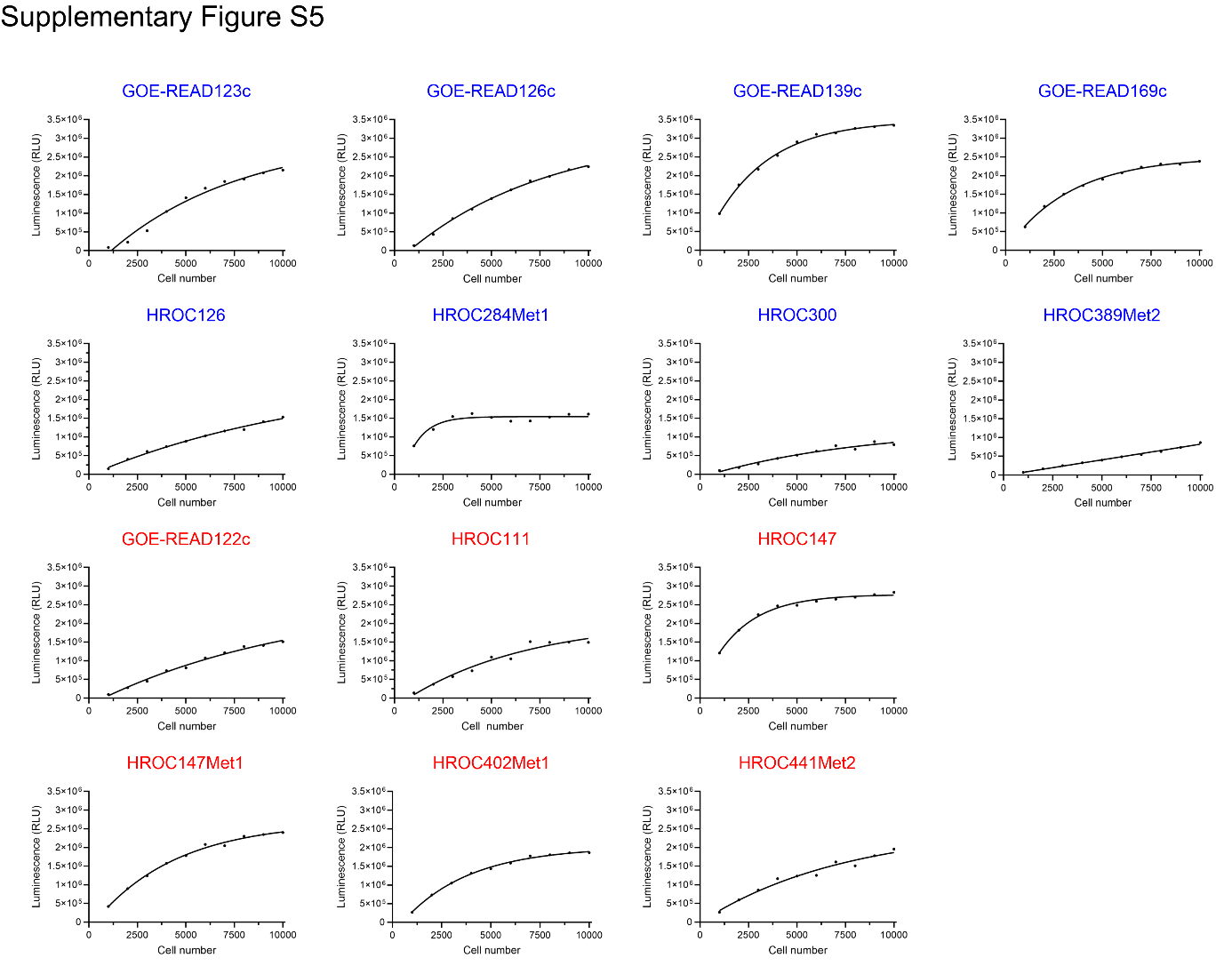


**Figure S5: Cell number establishment for drug screening experiments.**

Luminescence values measured by ATP-based cellular viability assay 96 hours post-seeding, across a range of increasing cell numbers per well, starting from 1000. Blue: (chemo)irradiation (IR)-sensitive PDCLs and red: IR-resistant PDCLs.

RLU, relative light units; IR, (chemo)irradiation.


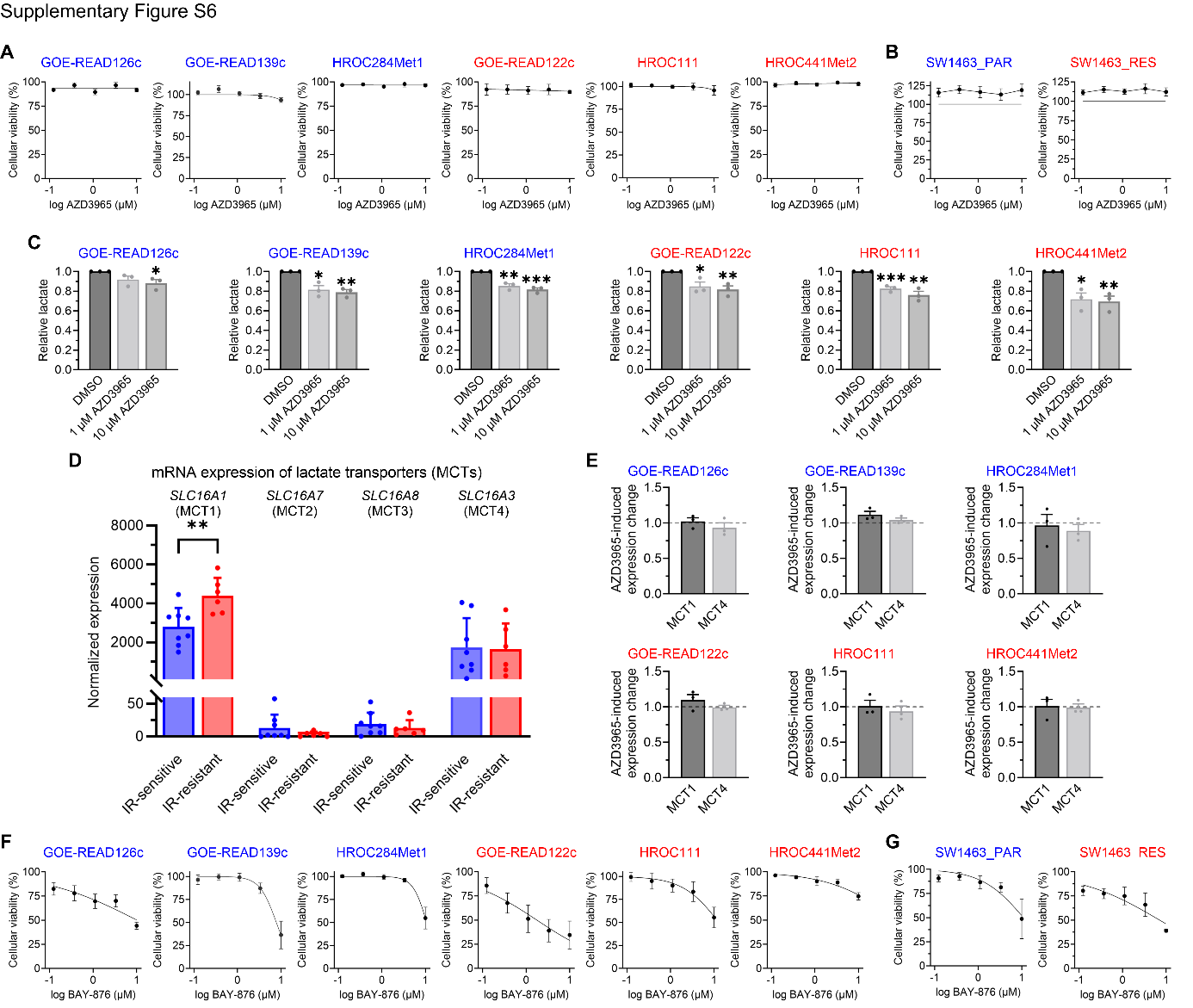


**Supplementary Figure S6: Functional validation experiments in AZD3965- and BAY-876-treated patient-derived cell lines.**

**A-B**) AZD3965-dependent dose-response curves of (**A**) (chemo)irradiation (IR)-sensitive (GOE-READ126c, GOE-READ139c, HROC284Met1) and IR-resistant (GOE-READ122c, HROC111, HROC441Met2) patient-derived cell lines (PDCLs) and (**B**) of IR-sensitive parental SW1463_PAR and IR-resistant SW1463_RES. Blue: IR-sensitive cell lines, red: IR-resistant cell lines. (**C**) Extracellular lactate levels relative to DMSO control in IR-sensitive (GOE-READ126c, GOE-READ139c, HROC284Met1) and IR-resistant (GOE-READ122c, HROC111, HROC441Met2) PDCLs after 72 h treatment with 1 µM or 10 µM AZD3965. Student’s ttest: *p < 0.05; **p < 0.01; ***p < 0.001. (**D**) Normalized mRNA expression (derived from RNA sequencing) of *SLC16A1* (encoding MCT1), *SLC16A7* (encoding MCT2), *SLC16A8* (encoding MCT3), *SLC16A3* (encoding MCT4) in IR-sensitive (blue) vs. IR-resistant (red) PDCLs. **p < 0.01. (**E**) qPCR analysis of *SLC16A1* (MCT1) and *SLC16A3* (MCT4) expression in IR-sensitive (GOE-READ126c, GOE-READ139c, HROC284Met1) and IR-resistant (GOE-READ122c, HROC111, HROC441Met2) PDCLs following 72 hours treatment with 10 µM AZD3965. Expression levels are calculated by 2^-ΔΔCT^ algorithm and normalized to DMSO-treated controls. **F-G**) BAY-876-dependent dose-response curves of (**F**) IR-sensitive (GOE-READ126c, GOE-READ139c, HROC284Met1) and IR-resistant (GOE-READ122c, HROC111, HROC441Met2) PDCLs and (**G**) of IR-sensitive parental SW1463_PAR and IR-resistant SW1463_RES. Blue: IR-sensitive cell lines, red: IR-resistant cell lines.

PDCL, patient-derived cell line; *SLC16A1*, solute carrier family 16 member 1; *SLC16A7*, solute carrier family 16 member 7; *SLC16A8*, solute carrier family 16 member 8; *SLC16A3*, solute carrier family 16 member 3; IR, (chemo)irradiation.
